# Application and Evaluation of a Multimodal Training on the Second Victim Phenomenon at the European Researchers’ Network Working on Second Victims Training School: Mixed Methods Study

**DOI:** 10.2196/58727

**Published:** 2024-08-30

**Authors:** Sofia Guerra-Paiva, José Joaquín Mira, Reinhard Strametz, Joana Fernandes, Victoria Klemm, Andrea Madarasova Geckova, Bojana Knezevic, Eva Potura, Sandra Buttigieg, Irene Carrillo, Paulo Sousa

**Affiliations:** 1 NOVA National School of Public Health Public Health Research Centre, Comprehensive Health Research Center NOVA University Lisbon Lisbon Portugal; 2 Alicante-Sant Joan Health District Alicante Spain; 3 Department of Health Psychology, Miguel Hernandez University Elche Spain; 4 Wiesbaden Institute for Healthcare Economics and Patient Safety, RheinMain University of Applied Sciences Wiesbaden Germany; 5 NOVA National School of Public Health, NOVA University Lisbon Lisbon Portugal; 6 Department of Health Psychology and Research Methodology, Faculty of Medicine, University of Pavol Jozef Šafárik Košice Slovakia; 7 Institute of Applied Psychology, Faculty of Social and Economic Sciences, Comenius University Bratislava Slovakia; 8 Department for Quality Assurance and Improvement in Health Care, University Hospital Centre Zagreb Zagreb Croatia; 9 Gesundheit Österreich GmbH, Bundesinstitut für Qualität im Gesundheitswesen Vienna Austria; 10 Department of Health Systems Management and Leadership, Faculty of Health Sciences,University of Malta Malta Malta

**Keywords:** patient safety, second victim, training, education, healthcare

## Abstract

**Background:**

Health care workers (HCWs) are often impacted by distressing situations during patient care and can experience the second victim phenomenon (SVP). Addressing an adequate response, training, and increasing awareness of the SVP can increase HCWs’ well-being and ultimately improve the quality of care and patient safety.

**Objective:**

This study aims to describe and evaluate a multimodal training organized by the European Researchers’ Network Working on Second Victims to increase knowledge and overall awareness of SVP and second victim programs.

**Methods:**

We implemented a multimodal training program, following an iterative approach based on a continuous quality improvement process, to enhance the methodology and materials of the training program over the duration of 2 years. We conducted web-based surveys and group interviews to evaluate the scope and design of the training, self-directed learning materials, and face-to-face activities.

**Results:**

Out of 42 accepted candidates, 38 (90%) participants attended the 2 editions of the Training School program. In the second edition, the level of participants’ satisfaction increased, particularly when adjusting the allocated time for the case studies’ discussion (*P*<.001). After the multimodal training, participants stated that they had a better awareness and understanding of the SVP, support interventions, and its impact on health care. The main strengths of this Training School were the interdisciplinary approach as well as the contact with multiple cultures, the diversity of learning materials, and the commitment of the trainers and organizing team.

**Conclusions:**

This multimodal training is suitable for different stakeholders of the health care community, including HCWs, clinical managers, patient safety and quality-of-care teams, academicians, researchers, and postgraduate students, regardless of their prior experience with SVP. Furthermore, this study represents a pioneering effort in elucidating the materials and methodology essential for extending this training approach to similar contexts.

## Introduction

### Background

During the care process, unexpected incidents may occur and result in either harm to patients (adverse events) or pose a risk to the health care system without directly impacting patient well-being (near misses) [1,2]. Approximately 1 in 10 patients is harmed in health care [3,4].

There is no doubt that the patient is the first victim of health care incidents. However, health care workers (HCWs) directly or indirectly involved can also experience the impact of these incidents. For this reason, they are often referred to as the second victims of adverse events.

According to a consensus-based definition, a second victim is “any healthcare worker, directly or indirectly involved in an unanticipated adverse patient event, unintentional healthcare error, or patient injury, and who becomes victimized in the sense that they are also negatively impacted” [5]. Physical consequences such as troubling memories, anxiety or concern, sleep disorders, and distress [6] are common reactions experienced by HCWs after patient safety incidents. Moreover, emotional responses such as anger toward themselves, regret or remorse, fear of future errors, embarrassment, and guilt are HCWs’ frequent reactions after health care incidents [6]. These consequences are associated with a decrease in work satisfaction, loss of confidence in their own abilities [7], turnover intentions, absenteeism [8], and even suicide in the most severe cases [9].

Available data suggest that between 60% and 92% of HCWs become second victims at least once during their careers [10-14]. However, a large number of managers and HCWs do not know how to act after a patient safety incident or how to cope with the second victim phenomenon (SVP) in their health care institutions [15,16]. Therefore, it is a phenomenon that, despite being a problem that receives inadequate attention, is present in health care and needs action and visibility among HCWs, managers, and other stakeholders, including the scientific and academic communities.

Addressing an adequate response to the SVP can reduce distress, the emotional burden of HCWs [7,11,17], and the financial impact stemming from avoidable health care incidents [18] and increase the quality of care and patient safety [19,20]. This support is key in reducing risks of future adverse events in the health care system and contributing to patient safety [19]. Therefore, increasing awareness of the support programs and SVP among the health care community and providing adequate training on the topics of SVP and patient safety are considered essential elements to effectively reduce the impact of health care incidents and to support second victims when facing stressful events [21]. The need for comprehensive training on SVP is supported by existing literature [22,23]. However, to our knowledge, there are no multidisciplinary and multicultural trainings focused on increasing awareness of the SVP and support programs, regardless of the cultural, political, and legal limitations of each country. Although there are some institutional programs focused on training peer supporters and HCWs on the SVP, there is a lack of training focused on preventive actions involving clinicians, researchers, and academicians.

Previous research indicates that training programs focusing on patient safety and related subjects should incorporate a structured implementation strategy with diverse teaching modalities. This approach ensures the activation and retention of knowledge among participants [24]. It is well known that the combination of traditional and active learning strategies enhances the learning process by creating accessible, flexible, and risk-free learning conditions [25,26].

Although the use of web-based materials for clinical training is increasing, case-based discussion remains the most commonly used form of active learning in health science education and training [27]. The case study methodology facilitates an in-depth examination of practical events or phenomena, generating knowledge applicable in real-life scenarios. This approach effectively bridges theoretical learning with practical application, enhancing the integration of theory into practice [28].

Different elements of interaction and discussion should be included to stimulate reflection and critical thinking. A scoping review published in 2020 shows that interactive approaches that offer the possibility to apply theoretical content into practice and adapt to the local environment are the most preferred among the health care workforce [29]. This study also highlights that opportunities to network with colleagues and discuss the training content and experiences using a collaborative approach are valued [29].

Multidisciplinary and interdisciplinary teamwork has multiple benefits, for example, the reduction of interpersonal conflicts at work, the fostering of psychological safety and mutual support, and the increase of both HCW’s well-being and patient safety [30,31]. Furthermore, this type of approach is highly recommended for SVP training [21,32].

In this study, we used a quality improvement process to evaluate and enhance the methods and materials of the multimodal training course. Our approach was particularly inspired by the philosophy of the plan-do-study-act (PDSA) model to guide improvements [32,33]. This cycle evolved from the Deming wheel introduced in 1950 and has been widely used in medical education and training to improve the quality of health care [33]. This is an iterative model consisting of the cyclical application of 4 phases, as indicated by its name (PDSA). Its application involves defining objectives, targets, and methods of interventions (plan); implementing the intervention and carrying out the data collection (do); analyzing the results of the implementation and summarizing what was learned (study); and making the necessary adjustments for improvement (act) [34]. This approach allows the continuous optimization of the interventions based on the knowledge and experience acquired during each iteration, as it is expected to use the insights gained from the application of the PDSA cycle in subsequent cycles. Although there are variations of this quality improvement model, such as the plan-do-check-act cycle, the PDSA model prioritizes data analysis and summarization of insights gained from the implementation process (study), which is beneficial for evidence-based interventions.

### Objectives

We aim to describe and evaluate the development and application of a multimodal training focused on increasing the awareness and knowledge on SVP and support programs and decreasing the stigma associated with patient safety incidents in health care. Our ultimate focus is to provide a multicultural training by incorporating resources that are freely available on the web; easily accessible; and suitable for HCWs (regardless of their clinical setting and culture), leaders of quality and patient safety in the institutions, health care managers, health science researchers, academicians, and students.

### Problem Identification

Before the development of intensive training, we identified the main factors influencing the low level of awareness of the SVP and second victim support programs. Before the development of the training, the European Researchers’ Network Working on Second Victims (ERNST) had been discussing how to address the lack of awareness of SVP and has invested in publishing evidence to increase knowledge in this area. This network was created to increase the resilience of the health care workforce in stressful situations, such as patient safety incidents. It is focused on facilitating discussions and sharing scientific knowledge, perspectives, and best practices focused on the SVP.

In Textbox 1, we describe the main causes influencing the lack of awareness of SVP and second victim support programs. They emerged from discussions within the ERNST group directly involved in the development of the educational materials for the multimodal training. This guided the planning and development of the multimodal intensive training.

**Factors that contribute to the low level of awareness and knowledge of the second victim phenomenon (SVP) and support programs.**

**Category and identified problem**

- People
  Lacking recognition of the problem by clinical leaders does not facilitate the prioritization of initiatives to promote the prevention of SVP in health organizations [16,35,36].
  Turnover and absenteeism after patient safety incidents [8] do not allow the clinical team to effectively support second victims and be aware of this problem.
  Clinicians often do not actively seek support when struggling with distressing situations [35].
- Communication
  Lack of dissemination of the problem among the clinical teams and health care community, which contributes to lack of information among health care workers (HCWs) [37-39]
- Culture
  Blame culture [40-42]
  Stigmatization of health care incidents [40-42]
  Lack of sensitization of second victim support initiatives in health organizations strongly influences the organizational culture, often fostering a climate of silence concerning health care incidents and distressing situations [15,16,43].
- Structures
  Lack of structures formally prepared for supporting HCWs after patient safety incidents [35]
- Materials
  Limited access to training materials and protocols [36]
- Training
  Limited investment in education and training on SVP in health care sciences curriculum [22,23]

## Methods

### Overview

In this study, we used a mixed methods design rooted in the quality improvement principles of planning, acting, evaluating, and adjusting to facilitate the ongoing enhancement of the intensive training method across a span of 2 years. This methodology was executed in 2 iterations of the face-to-face ERNST Training School program. The training falls within the framework of COST Action 19113 (2020 to 2024).

We applied the quality improvement process in the first face-to-face ERNST Training School in Zagreb, Croatia, in September 2022 and subsequently adjusted it in the second Training School in Wiesbaden, Germany, in October 2023. The dates of the face-to-face training were chosen according to the local organizing team’s availability, accessibility of resources, and COST Action orientations. After applying the design in the first edition of the Training School, we evaluated the materials, the design, and activities and reviewed the scope of the training to make the necessary improvements for the second edition of the Training School.

The design and materials of the Training School were developed and peer reviewed by the ERNST and the Training School team. During the Training School, these were evaluated by the trainees using web-based surveys and by conducting group interviews. Moreover, the ERNST organizing team also made daily follow-ups of the activities during the Training School, aiming to promptly address minor limitations or issues and offer constructive feedback on the progress of the work.

The ERNST Training School was promoted on the COST Action’s website, social media channels, ERNST website, ERNST newsletter, and trainers’ university intranets.

### Ethical Considerations

All the collected data were fully and irreversibly anonymized, and all participants were previously informed about the study objective and agreed with its terms and conditions. The study has been submitted to the National School of Public Health Ethical Commission and has received the institutional review board exemption (n1_2204).

### Description of the Training School: Objectives and Organizing Team

The main aim of the intensive training was to increase HCWs’ awareness and knowledge of the SVP and second victim support programs.

The organizing team was composed of a multicultural and multidisciplinary group of experts actively working on SVP research and with a background in health science. All the team members were part of the ERNST—COST Action CA19113.

All trainers had previous experience in education. The team comprised the Training School coordinator, local organizer team, and trainers. All trainers followed the common values of the Training School and were committed to the following:

- Guide trainees to achieve the learning goals of the case studies
- Support trainees on how to use the supporting resources
- Support trainees during the entire Training School (eg, tips, bibliography, and working with the training manual)
- Motivate trainees to actively participate in the Training School activities

In the following sections, we will describe the participants’ recruitment process and design and materials of the Training School.

### Recruitment of the Participants

The promotion of the Training School was achieved by dissemination through the ERNST website and the mailing list of ERNST members. Applications were evaluated by 2 independent members of the organizing team, applying criteria established by the COST Association rules (Vade mecum). Motivational letters and curricula vitae were required for the recruitment of the participants. The Training School was open to HCWs, health care managers, professionals working in quality of health and patient safety departments, academicians, researchers, and students of health sciences. Applications were only considered if participants had clinical or research experience in the field of health care. Previous work experience on SVP and patient safety or other topics related to quality of care was recommended. The participants were expected to be proficient in both written and spoken English.

### Training School Design

#### Overview

The Training School focused on 2 main strategies: self-directed learning (10 hours approximately) and face-to-face learning activities (approximately 3 working days, with a total of 24 hours of work).

The Training School predominantly focused on active learning methods. This included the application of case-based [44] and cooperative learning strategies [44] during the face-to-face intensive training and the use of web-based self-directed resources to support the learning process.

#### Self-Directed Activities

Self-directed training was voluntary and included web-based resources (eg, podcasts, YouTube [Google LLC] videos, training manual, and website) to support the trainees in their knowledge of the SVP and patient safety concepts as well as second victim support programs. The web-based resources were distributed 3 months before the Training School, allowing participants ample time to familiarize themselves with the topic and prepare for the intensive training.

#### Face-to-Face Learning Activities

Throughout the intensive training at the Training School, activities comprised establishing the theoretical foundation, engaging in group discussions, and interacting with real cases of the health care practice. The theoretical foundation was delivered through 2 main lectures centered on the fundamentals of the SVP and support interventions.

Roundtable discussions were incorporated into the training to provide direct contact to participants with real-life experiences. HCWs who had encountered the SVP and leaders from second victim support programs were invited to facilitate discussions and enhance the understanding of the practical application of these programs.

All the activities described in this section were essential for participating in the discussion of practical cases in the form of case studies. The Training School programs from the first and second editions are presented in Multimedia Appendix 1.

### Case Studies

The case studies focused on fictional scenarios that mirrored real-world situations encountered in practice. The practical cases were tailored to address 3 distinct levels of action: the organizational level, the clinical team level, and the individual (second victim) experience level.

The case studies were designed with the objectives of raising awareness of the SVP and support programs, enhancing trainees’ abilities to recognize signs of SVP, managing such incidents within their institutions, and fostering blame-free attitudes toward implementing risk management strategies for patient safety incidents.

**Figure 1:**
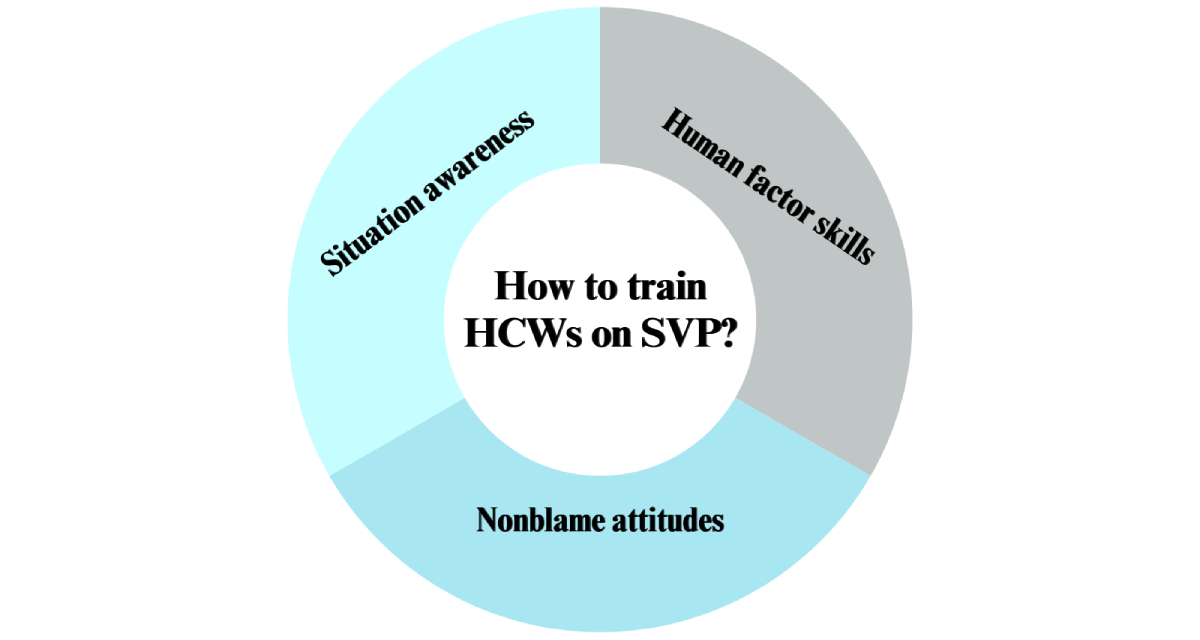
The 3 main domains to train health care workers (HCWs) on the second victim phenomenon (SVP) explored in the European Researchers’ Network Working on Second Victims case studies.

In Figure 1, the 3 main pillars of the case studies are described: situation awareness, human factors skills (dedicated to components of a system such as teamwork and communication to promote safety in health care and support HCWs’ work and well-being [45,46]), and nonblame attitudes toward error.

The scenarios included interdisciplinary responses to adverse events occurring in acute, primary, and ambulatory care settings, involving various actors in the care process.

In total, the intensive training included the discussion of 3 case studies. The scope of the discussion followed a pathway from a broader spectrum (organizational level) to more specific contexts (communitarian, primary, and acute care):

1. Developing and implementing a support program for HCWs involved in patient safety incidents
2. The path of a health care professional in the aftermath of a patient safety incident (involving community, primary care, and acute care)
3. Patient safety incidents in the hospital setting

All the case studies were developed by a group of experts from working group 3 and peer reviewed by some other elements of the same working group. Later, other members of the core group of the ERNST reviewed the overall content. All the involved members in the development of the case studies and peer review process were specialized experts working on quality of health and patient safety, psychologists, physicians, risk managers, and public health researchers.

During the Training School, the participants were divided into 5 working groups to explore the case studies. Each group was led by a qualified patient safety trainer with work experience on the SVP to facilitate the learning process and ensure that each working group achieved the learning goals (detailed in Multimedia Appendix 2). Working groups were given 1 hour for discussion and 8 to 10 minutes to present their main conclusions in a plenary session. The organization of working group members varied throughout the case studies to promote greater diversity in discussions and the sharing of experiences.

### Training School Materials

The multimodal educational training incorporates a variety of resources, such as audio files and self-paced learning materials, all aimed at nurturing critical thinking, enhancing decision-making abilities, and inspiring the generation of new ideas.

Each participant received a notebook that included the description of 3 case studies along with specific learning goals for the trainees (the notebook is presented in Multimedia Appendix 3). The participants of the first edition received a physical copy, while the participants of the second edition received it in PDF format for sustainability reasons.

The ERNST Training School Program included complementary web-based resources and activities to support the participants’ learning process. The complementary web-based resources were shared before the face-to-face Training School to establish common level of overall knowledge about the SVP and patient safety among the participants.

The web-based training manual was designed to provide comprehensive theoretical support to trainees regarding patient safety and the SVP. The various topics were categorized, allowing participants to explore them based on their individual interests and preferences.

On the ERNST website, participants had access to support videos featuring practical examples of the SVP within health care organizations as well as discussions on the fundamentals of patient safety and the SVP.

All these materials were developed by ERNST and peer reviewed. They were essential resources to complement the learning process and the discussion of practical cases in the form of case studies. The Training School resources are presented in Multimedia Appendix 3.

### Evaluation of the Training School

During the ERNST Training School, we applied web-based surveys using Google Forms (Google LLC) to evaluate the case studies’ methodology and to understand the participants’ profiles.

In total, we applied 6 web-based surveys:

- 1 survey for collecting demographic data of the participants
- 1 survey for investigating recent SVP experience, its prevalence, and support after the incident or stressful situation (adapted version of SeViD-II) [47]
- 3 surveys to validate ERNST Training School case studies after each case study activity
- 1 survey to evaluate the preconference implemented in the second edition of the Training School

All surveys are available in Multimedia Appendix 4.

Descriptive statistics was used to summarize the prevalence, and the Fisher exact test was used to compare differences between the 2 Training School editions. We used SPSS software (version 19.0.1.1; IBM Corp) to analyze the collected data.

On the final of each edition of the intensive training, we conducted group interviews to evaluate the overall organization and to gain insights into participants’ perceptions of the SVP following their participation in the Training School. During these group interviews, participants were given the opportunity to give recommendations for improvement for the next editions. Open-ended questions were analyzed using thematic analysis (questions are presented in Multimedia Appendix 5).

### Application of Continuous Improvement

After assessing the Training School materials, activities, and design in the first edition, we introduced changes in the second edition of the Training School. All changes were within the scope of the Training School.

In the quantitative analysis, we considered a consensus rate of ≥80% as indicative of an effective strategy or process. All the adjustments were agreed upon by the core organizing team, constituted by the Training School coordinator, Chair of ERNST, and local organizing team.

## Results

### Overview

In total, 20 participants from 11 countries and 22 participants from 14 countries were accepted in the first and second editions of the ERNST Training School, respectively. Participants from Austria, Croatia, Estonia, Germany, Italy, Portugal, Serbia, and Spain attended in both editions; those from Finland, Romania, and Ukraine only attended the first edition; and those from Azerbaijan, Bulgaria, Iceland, Malta, Moldova, and Turkey only attended the second edition.

In both editions, most attendants were female, accounting for 70% (14/20) of the accepted participants in the first edition and 82% (18/22) in the second edition. Furthermore, in both training editions, most of the attendants in the Training School were aged <40 years and had <5 years of work experience. In comparison with the first edition where 3 (15%) out of 20 were aged between 40 and 60 years old, a higher number of individuals were aged between 40 and 60 years in the second edition (7/22, 32%). More detailed information is presented in Tables S1 and S2 in Multimedia Appendix 6.

The participants came from different professional backgrounds, including academia, hospitals, primary care, and the pharmaceutical field. In the second edition, there was a greater diversity in participants’ profiles, including individuals from mental health and patient safety agencies, as well as varying levels of experience. The professional profiles are described in further detail in Tables S2 and S3 in Multimedia Appendix 6.

Most of the participants from the first and second editions who attended the editions of the Training School lacked previous experience in patient safety (14/20, 70% and 13/20, 59%, respectively). Additionally, 59% (13/20) of the accepted participants in the first edition had limited involvement in second victim support initiatives or research projects around that topic. However, this percentage decreased in the second edition with only 23% (5/22) of the accepted participants having inexperience in this area (Table S4 in Multimedia Appendix 6).

In both editions, some of the participants withdrew before the Training School started. Specifically, 1 (5%) participant withdrew from the first edition, and 3 (15%) participants from the second edition. Ultimately, 19 participants joined each edition in both 2022 and 2023.

Before the intensive training, 60% (23/38) of the participants were aware of the term *second victim*. In fact, 50% (19/38) of all participants had personally experienced such trauma during their professional careers in health care. Further details are presented in Table S5 and S6 in Multimedia Appendix 6.

Of the group of participants who reported previously experiencing symptoms of SVP (19/38, 50%), 37% (7/19) of the cases were associated with aggressive behaviors of patients or relatives, 32% (6/19) were related to incidents that didn’t directly cause harm to the patient, 26% (5/19) were linked with incidents that cause harm to the patient harm, and (1/19, 5%) with unexpected death or suicide of a patient. More details are presented in Table 1.

In both editions, most participants (13/19, 68%) received informal support from colleagues, supervisors, family members, or friends. Other participants did not ask for help after the traumatic event (6/19, 32%). Most participants recovered within 1 month (8/19, 42%) or within a week (5/19, 26%). The results are presented in Table 1, and more detailed information about the profile of participants’ experiences of each edition is given in Tables S6 and S7 in Multimedia Appendix 6.

**Table 1:** Previous experience of second victim phenomenon (SVP) from both Training School editions.

Responses		Values, n (%)
**Did participants experience the SVP during their professional career in health care? (N=38)**		
	Yes, in 1 event	8 (21)
	Yes, in >1 events	11 (29)
	No	19 (50)
**Did this event (if >1, at least 1 of them) take place within the last 12 months?^a^** **(n=19)**		
	Yes	11 (58)
	No	8 (42)
**What kind of event was it?^a^** **(n=19)**		
	Incident without patient harm or near harm	6 (32)
	Incident with patient harm	5 (26)
	Aggressive behavior of a patient or relative	7 (37)
	Unexpected death or suicide of a patient	1 (5)
**Did you receive support from others during the event?^a^** **(n=19)**		
	No, although I have not asked for help	6 (32)
	Yes, from colleagues	8 (42)
	Yes, from supervisor	2 (10)
	Yes, from family and friends	3 (16)
**How long did it take you to fully recover from the event?^a^** **(n=19)**		
	<1 day	2 (10)
	Within a week	5 (26)
	Within 1 month	8 (42)
	Within 1 year	1 (5)
	>1 year	1 (5)
	Not fully recovered	2 (10)

^a^These questions only included participants who previously experienced the SVP.

### Evaluation of Training School Materials and Activities

#### Overview

The participants of both editions found the methodology of the Training School activities adequate for the learning process and suitable for clinical practice. In the subsequent section, we will outline the evaluation of the ERNST Training School methodology using a mixed methods approach as well as the evaluation of case studies through quantitative analysis. The SPSS output can be consulted in Multimedia Appendix 7.

#### Overall Evaluation of the Training School Methodology: Strong Points

After the ERNST Training School, participants reported an increased awareness and understanding of the SVP and its impact on health care:

I didn’t know that the impact of SVP was so serious - the number of second victims and also the burden for the overall health system.

Furthermore, they mentioned having gained more knowledge about the procedures to follow after patient safety incidents. The participants highlighted the importance of learning about the evaluation of second victim support programs.

The commitment of the leaders and organizing team, the interdisciplinarity, and the interaction between diverse cultures were highlighted as strong points in both editions:

It was a rich experience to network with colleagues from other settings and countries.

Trainers were well prepared, groups well organised and topics were clear.

The diversity of the materials was also a strong point of the training, and some expressions mentioned by the participants were as follows:

I enjoyed having diversity in learning methods.

I was never bored as we had lectures, discussions, interactions...

Participants’ rotation in the group activities was a very strong point. The close discussion in roundtables with a higher number of participants enriched the experience very much.

A consensus rate of ≥80% (among at least 15 participants in a total of 19) was indicative that case studies were an effective strategy or process, leading us to conclude that adjustments were unnecessary for the second edition. In the first edition, 85% (44/52) of the responses combined for the 3 case studies indicated that the method of working group discussions was adequate to achieve the learning goals; therefore, we found that this method was beneficial for the learning process.

The learning goals and content of the case studies were perceived as clear by most respondents, with 85% (44/52) and 89% (46/52) of the respondents agreeing on this, respectively. The scenarios given in the case studies were considered realistic in comparison to current health care practices in 89% (46/52) of the responses, and 81% (42/52) of the participants agreed that the knowledge obtained from the case studies had a positive impact on their daily practice.

In the first edition, 85% (44/52) of the responses indicated that participants would recommend the 3 case studies to their colleagues to learn more about the SVP.

More detailed information is presented in Tables S8-S11 in Multimedia Appendix 6.

#### Adjustments Made After the First Edition: Applied in the Second Edition

##### Supporting Resources

In the first edition of the ERNST Training School, participants suggested that complementary materials for case study discussions (such as notebooks, podcast episodes, and training manuals) could be provided before the face-to-face event. After receiving this feedback, the organizing team decided to send the notebook in PDF format, along with all other complementary materials (including the new resources) before the face to face edition.

While 78% (28/36) of responses indicated clarity regarding the supporting information of the case studies (explanations, examples, and other sources suggestions), upon reviewing the feedback, we recognized the need for improvement. Specifically, in supporting materials for case study 1, only 63% (12/19) of the participants considered the supporting information to be clear.

Therefore, we revised and supplemented these support resources to enhance the learning experience for participants. We launched 5 podcast episodes to describe the interventions included in case study 1 in more detail (the link to the 5 podcast episodes is presented in Multimedia Appendix 3). The second victim podcast is a resource publicly available on >10 web-based platforms and focuses on talking about practical examples of second victim support programs, support strategies, leadership commitment, and education. In each podcast, the host invites a guest to talk about a second victim support program. While 58% (7/12) of the participants fully agreed that the podcast episodes were useful for the case study discussion in the first edition, after that adjustment, 82% (14/17) of the participants from the second edition who had listened to the podcast episodes fully agreed that the podcast was useful for the case study discussion (Table S12 in Multimedia Appendix 6).

In the second edition of the Training School, 20-minute videos covering the basics of patient safety and crew resource management were introduced to provide some fundamental knowledge for those less familiar with the topics. As these videos were not directly related to the case studies and Training School activities, they were not quantitatively evaluated.

##### Activities

In the first edition, participants expressed that enhancing communication during icebreaker activities would improve interaction among them. Therefore, the icebreaker activities in the second edition incorporated *rotating* roundtable discussions. In the first round, participants shared their backgrounds with others, and in the second round, they presented the collective background of the group they belonged to previously. In the concluding group interview of the second edition of the Training School, participants rated the icebreaker activities as very positive, noting their effectiveness in promoting interaction among participants and fostering involvement with the entire group.

Although participants from the first edition expressed that “it could be important to include more legal background in the lectures,” we prioritized lectures focused on successful second victim support programs and other strategies to increase second victim awareness and peer support competencies. Moreover, across the different European countries, different regulatory and legislative aspects are applicable.

For the second edition of the Training School, we held a web-based preconference meeting 4 months before the face-to-face event. The preconference was a recommendation from the group interviews of the first edition (the qualitative feedback is detailed in Table S8 in Multimedia Appendix 6). After the preconference meeting, 95% (18/19) of the participants considered it useful and 74% (14/19) indicated that the content was clear. When asked about the duration, 63% (12/19) of the participants fully agreed and 32% (6/19) partially agreed that the time for the web-based event was adequate (for more information, refer to Table S13 in Multimedia Appendix 6).

According to the participants’ feedback, the virtual meeting helped them to understand the overall goals of the Training School (13/19, 68%), and it was deemed important to meet the trainers and other trainees beforehand (15/19, 79%). Participants reported feeling more involved in the Training School after the web-based event (12/19, 63%), gained more self-confidence after learning about the other participants’ profiles (2/19, 10%), and found it easier to follow the steps for e-cost reimbursement (9/19, 47%). In addition, participants reported increased motivation to attend the Training School after participating in the preconference (15/19, 79%; more information is presented in Table S13 in Multimedia Appendix 6).

##### Program and Schedule

In the second edition, we adjusted the schedule of the activities to better accommodate working group discussions. In the first edition, 67% (35/52) of the participants considered the time adequate for working group discussions, including the preparation for the working group presentations in the plenary session (23/52, 44%). To improve time management, the schedule was adjusted in the second edition to ensure adequate time for discussing the case studies and preparing the presentations. The case study discussions were scheduled as the final activities of the day to accommodate the groups’ preferences. After these changes, we found significant differences in participants’ evaluation between the 2 editions of the Training School (*P*<.001). Moreover, the participants highlighted the planning and schedule as strong points of the second Training School:

The way the schedule was planned – with more “active” sessions in the morning and group dynamics - was a strong point.

The roundtables with the experts were valued in both editions; however, in the second edition, it was mentioned that the duration should be extended to elaborate on some topics.

On the basis of the feedback from the first edition, an additional day was incorporated for Training School activities. It allowed the inclusion of more breaks and additional time to develop the activities. In the second edition’s feedback, the organization of the schedule and the inclusion of breaks throughout the day were appreciated by the trainees (Table 2). All the adjustments are summarized in Table 2.

**Figure 2:**
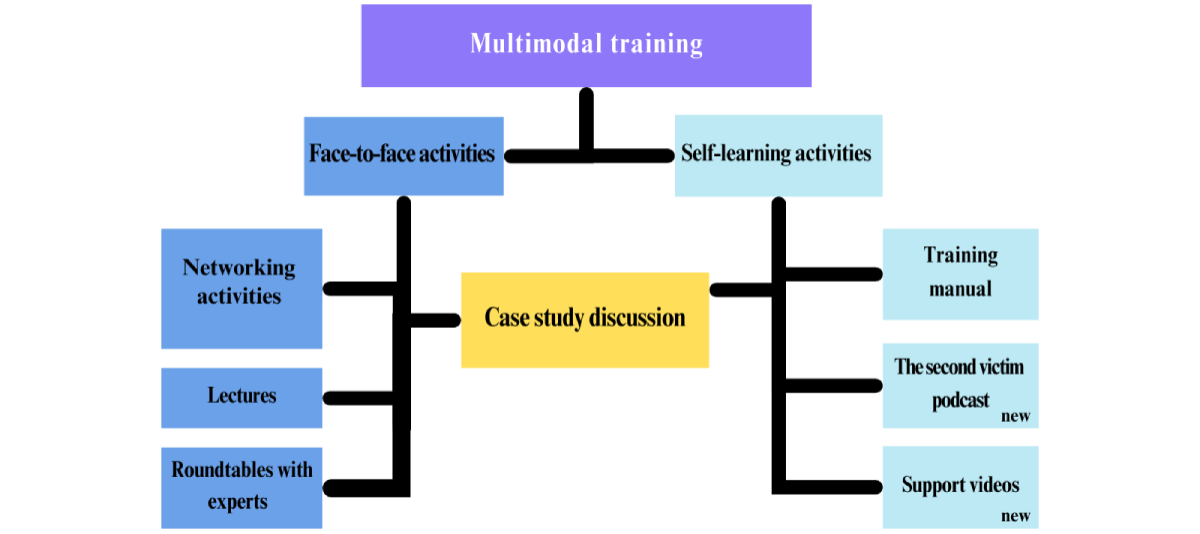
Methodological framework after the second edition of the Training School.

After making all modifications, the final Training School structure is presented in Figure 2. The resources included in the framework after the adjustments are marked in the figure as *new*.

**Table 2:** Summary of the overall results based on the application of the quality improvement process.

Main indications for improvement of the first edition	Adjustment applied in the second Training School edition	Overall results of the second edition
The training period should be longer because the activities’ schedule was very intense. The schedule should incorporate more breaks to understand the activity points and offer more time to develop the activities.	One day of intensive training was added. The schedule included more breaks. The activities ended earlier in the day.	The schedule was well received and praised by the participants. Breaks were valued by the participants.
The time for working group discussions and preparation of the presentation of the main conclusions was considered adequate in 67% (35/52) and 44% (23/52) of the responses, respectively. This indicates that the time for group discussion should be adjusted for the next edition.	The schedule was adjusted to allow more time for discussing the case studies and preparing the presentations. Most of the activities that included group discussions were scheduled for the end of the day.	The responses indicated that the time for discussion was adequate, as positive ratings increased from 67% (35/52) to 84% (47/56; *P*<.001). Furthermore, the positive ratings for the time for preparing the presentations of the main conclusions from the discussions significantly increased from 44% (23/52) to 86% (48/56; *P*<.001).
Supporting materials, such as the trainees’ notebooks, should be sent in advance in PDF format. The notebook was given to the trainees during the Training School as a physical copy.	All the complementary web-based resources were shared 4 months before the Training School. The notebook was provided in PDF format.	A physical copy of the notebook was preferred by some participants.
The supporting information to facilitate the discussion of case study 1 should be improved. This should include explanations, examples, and suggestions from other sources.	The complementary resources were reviewed. Two videos were included to complement the knowledge about patient safety principles and crew resource management. Five podcast episodes were included to describe the first case study’s interventions’ implementation, main achievements, and barriers.	Overall, participants appreciated the variety of learning materials. While not statistically significant, there was an increase in the proportion of participants who fully agreed that the supporting information for the case study 1 discussion was useful, rising from 63% (12/19) to 72% (13/18) in the second Training School edition (*P*=.48).
The participants expressed a desire for more information about the backgrounds of the trainers before the face-to-face Training School. A virtual preconference meeting was recommended to support trainees with logistics before the Training School and to serve as their initial contact before the face-to-face event.	A virtual preconference meeting was held. The event took place 4 months before the face-to-face Training School. It focused on the introduction of the trainers and trainees, the Training School methodology, program, and materials. In addition, it offered information about rules and organizational issues.	Participants valued the virtual preconference meeting. In total, 95% (18/19) of the participants fully agreed that the preconference meeting was useful. However, only 63% (12/19) of the participants deemed the duration of the meeting adequate, as some mentioned that 2 hours was too long for a working day.
It was recommended to create a web-based group (eg, LinkedIn; Microsoft Corp) or to share the email addresses of the participants to connect after the Training School.	After all the participants agreed to share their email addresses, the organizing team shared the contact list with all trainees.	No data were available about this topic.
The participants shared that more communication during the icebreaker activities would improve the participants’ interaction.	In the second edition, the icebreaker activities included *rotating* roundtables, where participants shared their backgrounds with others in the first round, and in the second round, they were supposed to present the overall background of the group they were in before.	The participants found the icebreaker activities very useful for participants interaction and involvement with the overall group.
In the first edition, participants expressed that it could be important to include more legal background in the lectures. We prioritized lectures focused on successful second victim support programs and other strategies to increase second victim awareness and peer support competencies.	The organizing team did not include a lecture focused on legal topics of SVP^a^ because legal frameworks differ from country to country.	In the second edition, the participants highlighted the need for legal background once again.

^a^SVP: second victim phenomenon.

## Discussion

### Principal Findings

In this study, we discovered that applying a quality improvement process contributed to enhancing the methodology and training materials of the intensive training program focused on increasing the knowledge and overall awareness of the SVP and second victim support programs.

Currently, there are no free, validated materials to debate the SVP in a multiprofessional and multicultural context. After applying the quality improvement process, we found that the educational materials are appropriate for replication in other similar trainings. Moreover, it is suitable for HCWs, clinical and patient safety and quality-of-care teams, academicians, researchers, and postgraduate students, regardless of whether they have previously experienced the SVP or not.

These materials are in line with the objectives set by the Patient Safety and Quality of Care Working Group [48] and the World Health Organization Global Action Plan for Patient Safety 2021 to 2030 [49]. Particularly, they align with strategic objective 5 of the World Health Organization Global Patient Safety Action Plan 2021 to 2030 [49], which focuses on inspiring, educating, and building skills and protecting health workers to contribute to the design and delivery of safe care. The SVP continues to be overlooked in medical and nursing curricula, and there is significant variability in the extent and quality of patient safety implementation in graduate education [23]. This intensive training could contribute to bridging the gap between curricula and practice by addressing the growing need to emphasize the effects of SVP in health sciences education.

One of the main strengths of this training lies in the integration of diverse European cultures and health care professions, fostering an interdisciplinary approach. Furthermore, the nature of this postgraduate continuing education program proportionated sharing a variety of practical insights by the participants, which proves beneficial in bridging the gap between various medical disciplines, theoretical knowledge, and real-world application. This facilitates its adaptation to other European multidisciplinary and interdisciplinary trainings focused on similar topics. In this sense, countries can find similar solutions to common problems related to HCWs’ support after traumatizing events.

The importance of interdisciplinarity in health science training is well recognized and recommended to improve the quality of care [50-52]. Research suggests that multidisciplinary collaboration enhances patient safety, particularly by reducing the occurrence of communication misunderstandings and decreasing the number of medical errors and associated complications [50,52]. Furthermore, it results in greater work satisfaction, increases HCWs’ productivity and creativity, enhances well-being, and reduces the risk of developing burnout [50,52].

The overall feedback regarding the Training School was positive in both editions, and the application of the quality improvement process facilitated adjustments in various aspects related to the training activities, self-directed resources to support the learning process, and the training program itself. In fact, the modifications made to the program, coupled with the extension of the activities’ duration, significantly improved the participants’ satisfaction. This was particularly evident in the increased time allocated for group discussions and for preparing presentations summarizing the key conclusions from group debates of the case studies.

Overall, most participants found the methodology adequate for the learning process and valued the commitment of the trainers in providing the best learning experience. The evidence indicates that the application of student-centered approaches and active learning methods enhances the learning experience [27] and is greatly appreciated in the training of health care disciplines [29]. Furthermore, a face-to-face format remains a valid approach in an era of emerging technologies, especially for case study discussions, roundtables with experts from clinical practice, and networking activities. Small group discussions are proven to have a greater impact on critical thinking, retention, and synthesis of knowledge compared to traditional lecture-based teaching, where information flow is predominantly unidirectional from the educator to the student [44,53,54]. These active learning activities require that the learners understand and use the knowledge derived from their educational experience to “construct” the learning process [27].

Furthermore, evidence indicates that using a combination of multiple learning formats and sensory sources (known as multimodal learning) activates cognitive functions and enhances engagement and interaction in the learning process. This is particularly dependent on how closely the learning content aligns with real-world scenarios [24,25,55,56]. In our study, the results from the applied surveys indicate that participants from both editions of the Training School valued the diversity of materials and the realistic scenarios presented in the case studies.

According to the Framework for Action on Interprofessional Education and Collaborative Practice, developing interprofessional education curricula should involve staff from different faculties, work settings, and locations [50]. At the ERNST Training School, participants had the opportunity to engage with individuals from diverse backgrounds and perspectives of the multidisciplinary organizing team. Moreover, one of the positive aspects pointed out by the participants from both editions was the commitment of the leaders and trainers to provide the best learning experience. Evidence shows that the leadership and behavior of clinical educators influence the learning experience [57-59]. It was found that regular feedback from trainers, which emphasizes the autonomy of the learning process and sets clear expectations, contributes to a psychologically safe environment grounded in principles of trust and nonjudgment. This, in turn, cultivates a sense of belonging and agency among participants [57,58]. In this training, the establishment of clear learning goals was one of the priorities to guide the training process while respecting the autonomy of the trainees. Another positive aspect was the rotating system of roundtables, which enabled the participants to directly interact with different educators and trainees. A supportive learning environment, which encourages interactions with both trainers and peers and offers a structured learning approach to guide the training process, is an essential factor for facilitating a productive learning experience [60].

In both editions, the participants emphasized the importance of exploring legal frameworks around the SVP. While acknowledging its importance, we chose not to cover this topic in the current training due to the diverse regulatory and legislative backgrounds across various countries. Nonetheless, this content may be incorporated into future SVP sessions. Furthermore, participants suggested establishing networking groups to stay in touch after the training. This highlights the preference for peer support solutions in the area of SVP [61].

Ultimately, this training program can address the need to raise the awareness of multiple stakeholders around the SVP and contribute to action in various areas of application. This study can serve as a valuable tool for enhancing competencies in managing SVP, and the resources are available on the web and can be easily reproduced.

### Limitations

Although we applied a rigorous methodology that was suitable to respond to the multiprofessional needs, it is important to acknowledge that the sample of participants was limited, which might influence the overall evaluation of the program. Therefore, we recommend that future interventions should include a broader range of participants, ensuring that it aligns with the format and adequacy of the training.

In addition, it is crucial to emphasize the significance of the diverse participant profiles observed between the first and second editions of the Training School. The potential variations in assessments resulting from participants’ diverse backgrounds and work experiences may pose limitations. However, our findings suggest that this diversity can also be leveraged as an asset to enhance materials by incorporating insights from a broader spectrum of profiles. Another limitation is that, in this study, we were unable to connect participants’ profiles with their responses, as the web-based surveys were anonymous. Therefore, we suggest that future studies should evaluate the connection between the trainees’ profiles and their assessments of training materials.

While overall satisfaction with the training program remained high, there was a decrease in the proportion of responses, indicating that participants would recommend the case studies to other colleagues from the first to the second edition. A similar observation was made when evaluating the learning goals and content of the case studies. Moreover, the scenarios of the case studies were rated less realistic by the participants of the second edition. Despite the observed differences in the evaluation of the case studies, the difference between both editions was not statistically significant.

In addition, it is important to highlight that all the supporting materials were provided on an optional basis for preparation for the Training School, which may influence the accuracy of the materials’ assessment.

### Conclusions

We conclude that this training is suitable for the health care community, including HCWs, clinical managers, patient safety and quality of health leaders in the institutions, researchers, academicians, and postgraduate students in health sciences.

This study describes the first applied multimodal training to increase awareness and knowledge about the SVP and second victim support programs and can be easily reproduced using the web-based resources available on the ERNST website, after contacting the ERNST Training School team. This intensive training addresses the need for education and training on SVP and contributes to bridging the gap between curricula and practice by providing tested and peer-reviewed training resources.

The application of quality improvement was useful to improve the quality of training, materials, activities, and the program. Its implementation resulted in improvements in participants’ satisfaction, particularly regarding the duration of activities, time for discussion with peers, and organization of the program.

In summary, the application of a student-centered approach, incorporating both traditional and active learning strategies, was beneficial for the SVP training program. Participants in the ERNST Training School highlighted the opportunity to engage in interprofessional training with individuals from various countries, along with the diversity of active learning methods, as some of the strongest aspects.

This training is reproducible in different health care contexts; however, further research is recommended to study the use of this type of training in a more extensive range of participants.

## Appendix

Multimedia Appendix 1 Detailed programs of the first and second editions of the European Researchers’ Network Working on Second Victims Training School.

Multimedia Appendix 2 Learning goals of the European Researchers’ Network Working on Second Victims Training School’s case studies.

Multimedia Appendix 3 The European Researchers’ Network Working on Second Victims Training School resources.

Multimedia Appendix 4 Web-based surveys applied in the European Researchers’ Network Working on Second Victims Training School.

Multimedia Appendix 5 Group interview guide.

Multimedia Appendix 6 Complementary tables presenting web-based survey results.

Multimedia Appendix 7 SPSS software (version 19.0.1.1; IBM Corp) output.

## Abbreviations

ERNST: European Researchers’ Network Working on Second Victims
HCW: health care worker
PDSA: plan-do-study-act
SVP: second victim phenomenon

## Data Availability

All data generated or analyzed during this study are included in this published article (in the multimedia appendix files).
