# Supplementary material for: Application and Evaluation of a Multimodal Training on the Second Victim Phenomenon at the European Researchers’ Network Working on Second Victims Training School: Mixed Methods Study
Source: JMIR Form Res. 2024 Aug 30;8:e58727. doi: 10.2196/58727 (PMC11418314; doi:10.2196/58727)
Supplement: Multimedia Appendix 1 [file formative_v8i1e58727_app1.docx]

## Multimedia Appendix 1

**Detailed programs of the first and second editions of the European Researchers’ Network Working on Second Victims Training School.**

**1^ST^ EDITION OF THE TRAINING SCHOOL**

**Venue:** University Hospital Centre Zagreb, Department for Psychiatry and Psychological Medicine, 1^st^ floor

**Address:** Kispaticeva 12, 10 000 Zagreb, Croatia

**TRAINERS:** Bojana Knejevic (BK) , Joana Fernandes (JF), José Joaquín Mira Solves (JMS), Reinhard Strametz (RS) Sandra Buttigieg (SB), Sofia Guerra Paiva (SGP)

**Colors guide**

**PLENARY SESSION**

**WORKING GROUP SESSION**

**VALIDATION PROCESS ACTIVITY**

| **Schedule** | |
| --- | --- |
| 9.00-9.20 | Registration |
| 9.20-10.00 | **Welcome session**  Host TS organizer (BK), Host institution leader (), ERNST Chair and Vice Chair (JMS, RS) |
| 10.00-10.45 | **Introduction of the TS Materials and Activities**  TS Coordinator (SGP) |
| 10.45-11.00 | Coffee break |
| 11.00 -12.00 | Group dynamic activities |
| 12.00 - 13.00 | Free time to explore the TS materials |
| 13:00-14.30 | Lunch break |
| 14.30-15.30 | **PLENARY SESSION 1 – Introduction of Second Victims Phenomenon**  Prof Reinhard Strametz |
| 15.30-16.00 | Q & A moment |
| 16.00-17.00 | Going to the City Center |
| 17.00-20.00 | Organized tour to visit Zagreb (optional) |
| 20.00 | Dinner (optional) |

**Monday, 3^th^ October 2022**

**Tuesday, 4th October 2022**

| **Schedule** | |
| --- | --- |
| 9.45- 10.50 | **PLENARY SESSION 3-** **Sharing experiences**  Trainees’ presentation about their experience in second victim support programs |
| 11.00-11.20 | Coffee break |
| 11.20- 12.30 | **Exploring Case study 1 -** working group session |
| 12.30-13.30 | **Exploring Case study 1 –** plenary discussion |
| 13.30-13.40 | Evaluation of the activity – Case study 1 |
| 13.40- 15.00 | Lunch Break |
| 15.00– 15.40 | **PLENARY SESSION 3-** **Sharing experiences**  Trainees’ presentation about their experience in second victim support programs |
| 15.40-15.50 | Break |
| 15.50 –17.00 | **Exploring Case study 2 -**working group session |
| 17.00- 18.15 | Presentation of main points of the round table |
| 19.00 | Dinner (optional) |

**Wednesday, 5^th^ October 2022**

| **Schedule** | |
| --- | --- |
| 9.00 – 10.00 | **Exploring Case study 3** |
| 10.00–10.45 | Presentation of main points of the round table |
| 10.45- 11.15 | Coffee break |
| 11.15 –12.00 | Roundtables with healthcare professionals |
| 12.00-13.00 | Overall discussion of the Training School in groups |
| 13.00- 13.30 | **Closing session**  TS Coordinator (SGP), Host TS organizer (BK), ERNST leader (JMS) |

**2^ND^ EDITION OF THE TRAINING SCHOOL**

**Title of the program:** Second victim phenomenon and support strategies for healthcare workers

**Venue:** Wiesbaden Business School

**Address:** Bleichstraße 44, 65183 Wiesbaden, Germany

**Trainers:** Jose Joaquin Mira, Reinhard Strametz, Sandra Buttigieg, Bojana Knezevic, Sofia Guerra Paiva, Joana Fernandes

**PLENARY**

**CASE STUDY SESSION**

**VALIDATION**

**Monday, 18^th^ September , 2023**

| **Time** | **Topic** |
| --- | --- |
| 8:15-8:30 | Registration |
| 8.30-9:00 | Welcome session (Hosts of the Training School, Chair of the ERNST, Training School Coordinator) |
| 9:00-9.30 | - Introduction of program of TS  - Brief introduction of the Trainers  - Presentation of the TS learning goals  - Presentation of the validation process of the TS materials |
| 9.30-11:00 | Trainees and Trainers roundtables and group dinamincs |
| 11:00 -11:30 | Coffee break |
| 11:30-12:30 | **PLENARY SESSION 1 – Introduction of Second Victim Phenomenon**  Reinhard Strametz |
| 12:30-13:00 | Q & A moment |
| 13:00-14:30 | Lunch break |
| 14:30-15:30 | **PLENARY SESSION 2 – Second victims support programs**  José Joaquin Mira |
| 15:30-16:00 | Q & A moment |
| 16:00 – 17:00 | Explore Training School materials and evaluate them |
| 18:30 | Meet for Dinner |

**Tuesday, 19th September 2023**

| **Time** | **Topic** |
| --- | --- |
| 8:30- 10:00 | **Exploring Case study 1 -** working group session |
| 10.00-10.30 | Coffee break |
| 10.30-11.15 | **Exploring Case study 1 –** plenary discussion |
| 11.15- 11.30 | Evaluation of the activity – Case study 1 |
| 11:30– 12:30 | **PLENARY SESSION 3** **– Talking with second victim support programs leaders**  Dominik Hinzmann  Eva Potura |
| 12.30-13.00 | Q&A moment |
| 13.00-14.30 | Lunch break |
| 14:30-17.00 | Roundtables with healthcare workers or /and risk managers and/or psychotherapists and/or hospital representatives |
| 18.30 | Dinner (optional) |

**Wednesday, 20^th^ September 2023**

| **Time** | Evaluation of the activity – Case study 2 |
| --- | --- |
| 8.30 – 10.00 | **Exploring Case study 2 -**working group session |
| 10.00-10.30 | Coffee break |
| 10.30-11.30 | **Exploring Case study 2 -** plenary discussion |
| 11.30 –11:45 | Evaluation of the activity – Case study 2 |
| 12.00-14.00 | Lunch break |
| 14.00- 15.00 | **Exploring Case study 3 -**working group session |
| 15.00-16.00 | **Exploring Case study 3 -** plenary discussion |
| 16.00-16.15 | Evaluation of the activity – Case study 3 |
| 18:30 | Meet for dinner |

**Thrusday, 21^st^  September 2023**

| **Time** | **Activity** |
| --- | --- |
| 8.30 – 10.00 | “Speakers corner” - (trainees posters presentation)  – e.g. Legal, economic, psychological, human factors, etc |
| 10:00-10:30 | Coffee break |
| 10:30-11h30 | Evaluation of the Training School |
| 11:30- 12:00 | Closing session |
| 12:00- 13:00 | Lunch break (lunch box) |
| 13:00- 19:00 | WIESBADEN TOUR (surroundings) |
| 19:00 | Dinner |
