# Supplementary material for: Application and Evaluation of a Multimodal Training on the Second Victim Phenomenon at the European Researchers’ Network Working on Second Victims Training School: Mixed Methods Study
Source: JMIR Form Res. 2024 Aug 30;8:e58727. doi: 10.2196/58727 (PMC11418314; doi:10.2196/58727)
Supplement: Multimedia Appendix 2 [file formative_v8i1e58727_app2.docx]

## Multimedia Appendix 2

**Learning goals of the European Researchers’ Network Working on Second Victims Training School’s case studies.**

| Learning goal: |
| --- |
| To describe different educational strategies to create awareness about second victim phenomenon. |
| To discuss why red flag experience can increase the awareness of patient safety issues and second victims’ problem. |
| To increase knowledge about creating awareness of second victim phenomenon. |
| To identify relevant stakeholders and justify their importance when preparing successful interventions. |
| To describe the potential facilitators for a second victim support programme implementation. |
| To identify what should be the steps for a second victim support programme preparation in a healthcare institution. |
| To identify what should be the steps for a second victim support programme preparation in a healthcare institution. |
| To describe the communication process and interaction among the healthcare teams implementing the support intervention. |
| To Identify the strengths and barriers of the support intervention (you can choose one intervention or more). |
| To identify indicators to assess the effectiveness of the support programme along the time and evaluation tools. |
| To identify the different strategies and pillars that will help to overcome barriers and make the support programme more sustainable overtime. |
| To identify different strategies to promote the support programme among the healthcare institution. |
| To identify different strategies to keep the teams motivated. |
| To list some of the emotions that the HCWs involved in patient safety incidents could experience. |
| To consider the potential consequences for the next patients when treated by a healthcare worker involved in a second victim |
| To describe some key factors that may influence each of the pathways of acting after patient safety (responding/ignoring the second victim phenomenon). |
| To describe potential barriers and how to overcome them (considering speaking-up; incident report; institutional support; open disclosure) |
| To describe how can the second victim experience influence the relations with the first and third victims and their outcomes (and vice-versa)? |
| To discuss if there are other patient safety subjects that might have influenced the second victim’ experience or that should be of interest to discuss in this context |
