# Supplementary material for: Application and Evaluation of a Multimodal Training on the Second Victim Phenomenon at the European Researchers’ Network Working on Second Victims Training School: Mixed Methods Study
Source: JMIR Form Res. 2024 Aug 30;8:e58727. doi: 10.2196/58727 (PMC11418314; doi:10.2196/58727)
Supplement: Multimedia Appendix 3 [file formative_v8i1e58727_app3.docx]

## Multimedia Appendix 3

**The European Researchers’ Network Working on Second Victims Training School resources.**

Podcast episodes: <https://cost-ernst.eu/podcast/>

Training manual: <https://trainingmanual.cost-ernst.eu>

Support videos on principles of patient safety:

<https://www.youtube.com/watch?v=VjuD8WZGLaM>

Support videos on crew resource management:

<https://www.youtube.com/watch?v=Oi1egJTQPic>

Training School notebook:

<https://cost-ernst.eu/training-school-notebook/>
