# Supplementary material for: Application and Evaluation of a Multimodal Training on the Second Victim Phenomenon at the European Researchers’ Network Working on Second Victims Training School: Mixed Methods Study
Source: JMIR Form Res. 2024 Aug 30;8:e58727. doi: 10.2196/58727 (PMC11418314; doi:10.2196/58727)
Supplement: Multimedia Appendix 4 [file formative_v8i1e58727_app4.docx]

## Multimedia Appendix 4

**Online surveys applied in the European Researchers’ Network Working on Second Victims Training School.**

**Questionnaire 1 – Preliminary questionnaire (1^st^ edition)**

[**https://docs.google.com/forms/d/1k6R848N2cEo8P1VnArWtwwubMpy5eSOphLLP9yJmfLU/prefill**](https://docs.google.com/forms/d/1k6R848N2cEo8P1VnArWtwwubMpy5eSOphLLP9yJmfLU/prefill)

**Questionnaire 1 – Preliminary questionnaire (2^nd^ edition)**

[**https://docs.google.com/forms/d/1UIZxUJJCjMxSd40oC4oR9_eMmA89XTb--5uL3o7UScI/prefill**](https://docs.google.com/forms/d/1UIZxUJJCjMxSd40oC4oR9_eMmA89XTb--5uL3o7UScI/prefill)

**Questionnaire 2 – Questionnaire on second victims (1^st^ and 2^nd^ edition)**

[**https://docs.google.com/forms/d/1vW1zWLgVjxLhm9I5HN0lZ4Nn1kZ3C6_ntSWgT_HmOus/prefill**](https://docs.google.com/forms/d/1vW1zWLgVjxLhm9I5HN0lZ4Nn1kZ3C6_ntSWgT_HmOus/prefill)

**Questionnaire 3 - Evaluation of Case Study 1 (1^st^ and 2^nd^ edition)**

[**https://docs.google.com/forms/d/1J0cqldEtp7MWKrPo9TEX4G4fTRka22iKSmx14XcAI_I/prefill**](https://docs.google.com/forms/d/1J0cqldEtp7MWKrPo9TEX4G4fTRka22iKSmx14XcAI_I/prefill)

**Questionnaire 4 – Evaluation of Case study 2 (1^st^ and 2^nd^ edition)**

[**https://docs.google.com/forms/d/16m9_AeT4kmJkOSox0XfI8Q5oC333wN86tSC3BLdkgUE/prefill**](https://docs.google.com/forms/d/16m9_AeT4kmJkOSox0XfI8Q5oC333wN86tSC3BLdkgUE/prefill)

**Questionnaire 5 – Evaluation of Case Study 3 (1^st^ and 2^nd^ edition)**

[**https://docs.google.com/forms/d/1rX7YNJdd1UE2kTZdAEN--obcRXj06qdP46qJbtyH2iE/prefill**](https://docs.google.com/forms/d/1rX7YNJdd1UE2kTZdAEN--obcRXj06qdP46qJbtyH2iE/prefill)

**Questionnaire 6 – Evaluation of the Pre Conference (2^nd^ edition)**

[**https://docs.google.com/forms/d/1u7X6axEZbY9G0vGdHI07G8d8otTUxpnPb54F2V6KhMM/prefill**](https://docs.google.com/forms/d/1u7X6axEZbY9G0vGdHI07G8d8otTUxpnPb54F2V6KhMM/prefill)
