# Supplementary material for: Application and Evaluation of a Multimodal Training on the Second Victim Phenomenon at the European Researchers’ Network Working on Second Victims Training School: Mixed Methods Study
Source: JMIR Form Res. 2024 Aug 30;8:e58727. doi: 10.2196/58727 (PMC11418314; doi:10.2196/58727)
Supplement: Multimedia Appendix 5 [file formative_v8i1e58727_app5.docx]

## Multimedia Appendix 5

**Group interview guide.**

Question 1 - What do you consider that were the strong points of the Training School?

Question 2- What aspects are in need for improvement?

Question 3- Do you have any recommendations for future editions?
