# Supplementary material for: Application and Evaluation of a Multimodal Training on the Second Victim Phenomenon at the European Researchers’ Network Working on Second Victims Training School: Mixed Methods Study
Source: JMIR Form Res. 2024 Aug 30;8:e58727. doi: 10.2196/58727 (PMC11418314; doi:10.2196/58727)
Supplement: Multimedia Appendix 6 [file formative_v8i1e58727_app6.docx]

**Multimedia Appendix 6**

**Complementary tables presenting web-based survey results.**

Table S1 – Participants’ demographics in the first and second face-to-face editions of the ERNST Training School

|  | 1st ERNST Training School Edition | | | 2nd ERNST Training School Edition | | |
| --- | --- | --- | --- | --- | --- | --- |
| Country |  | **N** | **%** | **Country** | **N** | **%** |
|  | Austria | 2 | 10% | Austria | 1 | 5% |
|  | Croatia | 3^1^ | 15% | Azerbaijan | 1 | 5% |
|  | Estonia | 1 | 5% | Bulgaria | 1 | 5% |
|  | Finland | 1 | 5% | Croatia | 2^1^ | 9% |
|  | Germany | 2 | 10% | Estonia | 3 | 14% |
|  | Italy | 1 | 5% | Germany | 2 | 9% |
|  | Portugal | 3 | 15% | Iceland | 1 | 5% |
|  | Romania | 2 | 10% | Italy | 1 | 5% |
|  | Serbia | 2 | 10% | Malta | 1 | 5% |
|  | Spain | 1 | 5% | Moldova | 1 | 5% |
|  | Ukraine | 2 | 10% | Portugal | 2 | 9% |
|  | Total | 20 | 100% | Serbia | 2 | 9% |
|  |  |  |  | Spain | 1 | 5% |
|  |  |  |  | Turkey | 3^2^ | 14% |
|  |  |  |  | Total | 22 | 100% |
| Gender | Female | 14 | 70% | Female | 18 | 82% |
| Age (Year) | <30 | 8 | 40% | <30 | 3 | 13.60% |
|  | 30-40 | 8 | 40% | 30-40 | 11 | 50% |
|  | 40-50 | 1 | 5% | 40-50 | 4 | 18,2% |
|  | 50-60 | 2 | 10% | 50-60 | 3 | 13.6% |
|  | >60 | 1 | 5% | >60 | 1 | 4.5% |

1. One participant withdrew from the Training School
2. Two participants withdrew from the Training School

| **1st ERNST Training School Edition** | | | | | |
| --- | --- | --- | --- | --- | --- |
| **Setting** | **N** | **%** | **Function** | **N** | **%** |
| Academic | 11 | 55 | Research ^1^ | 4 | 20% |
|  |  |  | PhD Student | 6 | 30% |
|  |  |  | Master | 1 | 5% |
|  |  |  | Educator | 1 | 5% |
| Hospital | 6 | 30 | Physicians | 3 | 15% |
|  |  |  | Resident Doctor | 2 | 10% |
|  |  |  |  |  |  |
| Primary Care | 2 | 10 | Physician | 1 | 5% |
|  |  |  | Resident Doctor | 1 | 5% |
| Pharmacy | 1 | 5 | Sales manager | 1 | 5% |
|  |  |  |  |  |  |
|  |  |  |  |  |  |
|  |  |  |  |  |  |
| Total | 20 | 100 | Total | 20 | 100 |
| Years of experience | | | | | |
| <5 years old | 10 | | | 50% | |
| 5-10 years old | 4^1^ | | | 20% | |
| 10-15 years old | 2 | | | 10% | |
| 15-20 years old | 1 | | | 5% | |
| >20 years old | 3 | | | 15% | |
| Total | 20 | | | 100% | |

Table S2- Professional profile of the participants from the 1^st^ edition

1. One participant withdrew from the Training School

Table S3- Professional profile of the participants 2^nd^ edition

| 2nd ERNST Training School Edition | | | | | |
| --- | --- | --- | --- | --- | --- |
| Setting | N | % | Function | N | % |
| Academic | 9 | 41 | Master Student | 1 | 5% |
|  |  |  | Phd student^1^ | 3 | 14% |
|  |  |  | Researcher^1^ | 3 | 14% |
|  |  |  | Educator | 2 | 9% |
| Hospital | 8 | 36 | Manager | 1 | 5% |
|  |  |  | Physician | 2 | 9% |
|  |  |  | Clinical Psychologist | 1 | 5% |
|  |  |  | Resident | 2 | 9% |
|  |  |  | Nurse^1^ | 2 | 9% |
| Primary Care | 2 | 9 | Doctor | 1 | 5% |
|  |  |  | Researcher | 1 | 5% |
| Mental Health | 1 | 5 | Counselor | 1 | 5% |
| Patient safety agency | 1 | 5 | Manager | 1 | 5% |
|  |  |  |  |  |  |
| Pharmacy | 1 | 5 | Regulator | 1 | 5% |
| Total | 22 | 100 |  | 22 | 100% |
| Years of experience | | | | | |
| <5 years old | 7^1^ | | | 32% | |
| 5-10 years old | 4^1^ | | | 18% | |
| 10-15 years old | 4^1^ | | | 18% | |
| 15-20 years old | 3 | | | 14% | |
| >20 years old | 4 | | | 18% | |
| Total | 22 | | | 100% | |

1. One participant withdrew from the Training School

Table S4- Previous experience with patient safety and second victim phenomenon

| PREVIOUS EXPERIENCE ON PATIENT SAFETY | | | | | PREVIOUS WORK EXPERIENCE ON SV SUPPORT INITIATIVES OR RESEARCH | | | | |
| --- | --- | --- | --- | --- | --- | --- | --- | --- | --- |
| 1st ERNST Training School Edition | | | 2^nd^ ERNST Training School Edition | | 1st ERNST Training School Edition | | | 2^nd^ ERNST Training School Edition | |
| Currently working on a patient safety position | 6 | 30% | 9 | 41% | Yes | 9 | 41% | 17 | 77% |
| Don't have previous experience on a patient safety position | 14 | 70% | 13 | 59% | No | 13 | 59% | 5 | 23% |
| Total | 20 | 100% | 22 | 100% | Total | 20 | 100% | 22 | 100% |

Table S5- Participants awareness of the term "second victim"

| 1^st^ ERNST Training School Edition | | | 2^nd^ ERNST Training School Edition | | |  | |
| --- | --- | --- | --- | --- | --- | --- | --- |
| Were participants aware of the term "second victim"^a^ prior to the intensive training? | | | | | | **Total** | |
| **Responses** | **N** | **%** | **Responses** | **N** | **%** | **N** | **%** |
| Yes | 12 | 63.2 | Yes | 11 | 57.9 | 23 | 60,5% |
| No | 7 | 36.8 | No | 8 | 42.1 | 15 | 39,5% |
| Total | 19 | 100 | Total | 19 | 100 | 38 | 100% |

a. Describes a person in healthcare who has been emotionally traumatised by an unexpected clinical event

Table S6 -Previous experience of second victim phenomenon lived by the participants from the 1^st^ edition and 2^nd^ edition

Table 5.1 - Previous experience of second victim phenomenon lived by the participants from the 1^st^ edition

| 1^st^ ERNST Training School Edition | | | | | | | | | |
| --- | --- | --- | --- | --- | --- | --- | --- | --- | --- |
|  | | Did participants experience the second victim phenomenon during their professional career in healthcare? | | | | | | | |
|  |  | Yes, in one event | | Yes, in more than one event | | No | | Total | |
|  |  |  |  |  |  |  |  |  |  |
|  |  | N | % | N | % | N | % | N | % |
|  |  | 4 | 21 | 5 | 26 | 10 | 53 | 19 | 100 |
|  |  |  |  |  |  |  |  |  |  |
| Did this event (if more than one, at least one of them) took place within the last 12 months? | Responses | N | % | N | % | N | % | N | % |
|  | Yes | 1 | 25 | 3 | 60 | - | - | 4 | 44 |
|  | No | 3 | 75 | 2 | 40 | - | - | 5 | 56 |
|  | Total | 4 | 100 | 5 | 100 | - | - | 9 | 100 |
| What kind of event was it? | Incident without patient harm/near harm | 2 | 50 | 0 |  | - | - | 2 | 22 |
|  | Incident with patient harm | 1 | 25 | 2 | 40 | - | - | 3 | 33 |
|  | Aggressive behaviour of a patient/relative | 1 | 25 | 2 | 40 | - | - | 3 | 33 |
|  | Unexpected death/suicide of a patient | - |  | 1 | 20 | - | - | 1 | 11 |
|  | Total | 4 | 100 | 5 | 100 | - | - | 9 | 100 |
| Did you receive support from others during the event. | No, although I have not asked for help. | 3 | 75 | - | - | - | - | 3 | 33 |
|  | Yes , from colleagues | - | - | 3 | 60 | - | - | 3 | 33 |
|  | Yes, from supervisor | - | - | 1 | 20 | - | - | 1 | 11 |
|  | Yes, Family and friends | 1 | 25 | 1 | 20 | - | - | 2 | 22 |
|  | Total | 4 | 100 | 5 | 100 | - | - | 9 | 100 |
| How long did it take you to fully recover from the event?  (In case of more than one, please consider the key event) | Less than one day | 1 | 25 | - |  | - | - | 1 | 11 |
|  | Within a week | - | - | 2 | 40 | - | - | 2 | 22 |
|  | Within one month | 2 | 50 | 3 | 60 | - | - | 5 | 56 |
|  | Within one year | 1 | 25 | - |  | - | - | 1 | 11 |
|  | Total | 4 | 100 | 5 | 100 |  |  | 9 | 100 |

Table S7 5.2- Previous experience of second victim phenomenon lived by the participants from the 2^nd^ edition

| 2^nd^ ERNST Training School Edition | | | | | | | | | |
| --- | --- | --- | --- | --- | --- | --- | --- | --- | --- |
|  | | Did participants experience the second victim phenomenon during their professional career in healthcare? | | | | | | | |
|  |  | Yes, in one event | | Yes, in more than one event | | No | | Total | |
|  |  |  |  |  |  |  |  |  |  |
|  |  | N | % | N | % | N | % | N | % |
|  |  | 4 | 21% | 6 | 32% | 9 | 47% | *19* | 100 |
|  |  |  |  |  |  |  |  |  |  |
| Did this event (if more than one, at least one of them) took place within the last 12 months? | Responses | N | % | N | % | N | % | N | % |
|  | Yes | 4 | 100 | 3 | 50 | - | - | 7 | 70% |
|  | No | - |  | 3 | 50 | - | - | 3 | 30% |
|  | Total | 4 | 100 | 6 | 100 | - | - | 10 | 100% |
| What kind of event was it? | Incident without patient harm/near harm | 1 | 25% | 3 | 50% | - | - | 4 | 40% |
|  | Incident with patient harm | 1 | 25% | 1 | 17% | - | - | 2 | 20% |
|  | Aggressive behavior of a patient/relative | 2 | 50% | 2 | 33% | - | - | 4 | 40% |
|  | Unexpected death/suicide of a patient | - |  | - |  | - | - | - |  |
|  | Total | 4 | 100 | 6 | 100 | - | - | 10 | 100 |
| Did you receive support from others during the event. | No, although I have not asked for help. | 1 | 25% | 2 | 33% | - | - | 3 | 30% |
|  | Yes , from colleagues | 2 | 50% | 3 | 50% | - | - | 5 | 50% |
|  | Yes, from supervisor | - |  | 1 | 17% | - | - | 1 | 10% |
|  | Yes, Family and friends | 1 | 25% | - |  | - | - | 1 | 10% |
|  | Total | 4 | 100 | 6 | 100 | - | - | 10 | 100 |
| How long did it take you to fully recover from the event? (in case of more than one, please consider the key event) | Less than one day | 1 | 25% | - | - | - | - | 1 | 10% |
|  | Within a week | 1 | 25% | 2 | 33% | - | - | 3 | 30% |
|  | Within one month | - | - | 3 | 50% | - | - | 3 | 30% |
|  | Within one year | - | - | - | - | - | - | - | - |
|  | More than one year | 1 | 25% | - | - | - | - | 1 | 10% |
|  | Not fully recovered | 1 | 25% | 1 | 17% |  |  | 2 | 20% |
|  | Total | 4 | 100 | 6 | 100 |  |  | 10 | 100 |

Table S8- Overall appreciation of the Training School

| Overall appreciation of the Training School | | | |
| --- | --- | --- | --- |
| Strong points | | | |
|  | | 1^st^ edition of the ERNST Training School | 2^nd^ edition of the ERNST Training School |
| Themes | Categories | Verbatim | Verbatim |
| Learning experience | Increased awareness on SV Topic | “I’ve gained awareness that the HCWs on my country are not different from those from another countries. But also, the diversity of departments/health care professionals present here *[being not different between themselves]*.”  “I wasn’t aware of the term. I didn’t know that the impact of the SVP was so serious- the number of SVs and the burden for the overall health system.” | - “I knew something about the term SV… however, I didn’t know about the support projects and research – and now I know we can use that.”  *“I’m more aware about the procedures that should be followed after the healthcare incidents”*  *“Understanding the importance of support programmes evaluation was particularly interesting for me and I will take that to my service.”*  *After this training I feel I want to do something in my service…preading awareness about SVP… and I’m thinking about implementing a peer support programme.* |
| Training School Organisation | Adequate Planning and Schedule | *“It was good to have time for discussion and Q&A moments during the plenary sessions”* | - *“The way the schedule was planned – with more “active” sessions in the morning and group dynamics - was a strong point”* - *“The preconference was on the right time”* - *“I found presentations in the morning a good point”* - *“Respected Schedule...”* - *“The way the training was organised turned the experience easy and very comfortable”* - *“It was important to have time for pause between the morning and afternoon and not to have activities until late”* |
|  | Commitment of the leaders/  Organising team | *“I felt that the leaders and organisation team were very committed to give us the best learning experience”*  *“Trainers were well prepared, groups well organised and topics were clear”* | - *“I felt very cared for by the organisation”* - *“I really appreciate the interaction with trainers and trainees”* |
| Training School Activities | Materials | *“work materials were very good – practical examples, realistic case studies”*  *“I think having different sources of information was important for the learning experience…”* | - *Case studies well chosen* - *“I enjoyed having diversity in learning methods. . I was never bored as we had lectures, discussions, interactions…”* |
|  | Organisation |  | - *“I really liked the preconference – where to stay/what to do…I felt that it was very welcoming” …“opportunity to get to know about the trainees.”* - *“I really liked the small roundtables, they were beneficial for me”* |
|  | Interaction | *“The experience was very enriching. I personally value the multiculturality and multi-professional exchange on topic that is similar to all of us”*  *“It was great to network with colleagues from other settings and countries”*  *- “For me the most important was the idea that we are not alone. I loved the multidisciplinary approach.”* | - *“I enjoyed having diversity in leaning methods. I was never bored as we had lectures, discussions, interactions…”* - *“I liked the dynamic of the activities”* - *Participants rotation in the activities was a very strong point. The close discussion in roundtables with a higher number of participants enriched the experience very much”* |
| To improve | | | |
| Trainng School Organisation | Planning and Schedule | *“Training should be 1 day longer”*  *“Maybe one day more to develop one extra activity”* |  |
|  |  | *-“It would be nice to smother the schedule agenda, have more pauses to understand the activity points, have more time to develop the activities”*  - *Some days we end up staying late in the activities … it was hard to concentrate after a full day of activities”* | - “*In the roundtables with experts, I would like to have more time to discuss with them and the group. I think it would be positive for deepen some topics…”* |
|  | -Complementary support activities and materials | *- “It would be important to have the notebook sent ahead”*  *- “I would like to have more information about the background of the Trainers, to better prepare the approach to the training”*  *- “having a preschool online meeting could be useful for the participants”*  *(…) also to have someone from COST explaining the CA and their opportunities (what is cost, Ernst, eCost, ORCID?) (…)”*  *- participants could write something about themselves and share a picture [to know something of each other before the arrival to the Training School]*  *- You could create some groups (eg linkedin) to connect all the team and participant after the Training School* | - *…The Preconference was important however I would do it in less then 2 hours, mostly because it happened on a working day…”*      - *It was difficult to use a small device in the case studies activities (eg, phone). – I prefer paper* - *For presentations, I would recommend a shared cloud to give access to all the group presentations* - *“After the Training School we could create a whatsapp group to keep in contact”* |
| Training School Activities | -Interaction | *- more room to discuss ideas, perceptions, etc with other trainees* |  |
|  | -Organisation | *- I felt that we should have more communication during the icebreaker*  *- you could modify the CS 1 organisation [specially the WG 5 felt that they were in the end of everything…]* | - *“It would be helpful to have the task to do always visible in the room (paper/projected)”* - *“I don’t have internet on my phone, this was a problem when answering the online questionnaires and accessing the online materials.”* |
|  | -Content | *-“It could be important to include more legal perspective in the lectures”*  *-“I would recommend to explore more practical examples”* | - *“Sometimes the learning confusing, sometimes no one was quite sure what was to be done” // “sometimes not clear”* - *“Perhaps to invite a legal expert developing work on the area”* |
|  | Space | *- more space (sometimes the noise from other groups in the same room was very loud – difficult to concentrate)* |  |

Table S9- Evaluation of cases studies from 1^st^ and 2^nd^ edition

Table 7.1 – Evaluation of cases studies 1^st^ edition

| Responses | Case study 1 | | Case study 2 | | Case study 3 | | Total | |
| --- | --- | --- | --- | --- | --- | --- | --- | --- |
| The leaning goals were clear | N | % | N | % | N | % | N | % |
| 1- fully disagree | - | - | - | - | - | - |  |  |
| 2- partly disagree | - | - | 1 | 5,88% | 1 | 6,25% | 2 | 3,85% |
| 3- partly agree | 3 | 15,79% | 2 | 11,76% | 1 | 6,25% | 6 | 11,54% |
| 4- fully agree | 16 | 84,21% | 14 | 82,35% | 14 | 87,50% | 44 | 84,62% |
| Total | 19 | 100% | 17 | 100,00% | 16 | 100% | 52 | 100,00% |
| The scenario was realistic comparing to the current healthcare practice | N | % | N | % | N | % | N | % |
| 1- fully disagree | - | - | - | - | - | - |  |  |
| 2- partly disagree |  | - | 1 | - | 1 | - | 2 | 3,85% |
| 3- partly agree | 2 | 10,5% | 1 | 11,8% | 1 | 6,25% | 4 | 7,69% |
| 4- fully agree | 18 | 94,7% | 15 | 88,2% | 15 | 93,75% | 46 | 88,46% |
| Total | 19 | 105% | 16 | 100% | 16 | 100% | 52 | 100,00% |
| The content of the case study was clear | N | % | N | % | N | % | N | % |
| 1- fully disagree | - | - | - | - | - | - |  |  |
| 2- partly disagree | - | - | - | - | - | - |  |  |
| 3- partly agree | 3 | 15,8% | 2 | 11,8% | 1 | 6,25% | 6 | 11,54% |
| 4- fully agree | 16 | 84,2% | 15 | 88,2% | 15 | 93,75% | 46 | 88,46% |
| Total | 19 | 100% | 17 | 100% | 16 | 100% | 52 | 100,00% |
| The supporting information of the case studies (explanations/examples/other sources suggestions) was clear. | N | % | N | % |  |  |  | % |
| 1- fully disagree | - | - | - | - | - | - |  |  |
| 2- partly disagree | - | - | 1 | 5,90 | - | - | 1 | 2,78% |
| 3- partly agree | 7 | 36,84% | - | - | - | - | 7 | 19,44% |
| 4- fully agree | 12 | 63,16% | 16 | 94,10 | - | - | 28 | 77,78% |
| Total | 19 | 100% | 17 |  |  |  | 36 |  |
| The knowledge obtained from the case study will (positively) affect my daily practice. | N | % | N | % | N | % | N | % |
| 1- fully disagree | - | - | - | - | - | - |  |  |
| 2- partly disagree | - | - | 2 | 12% | 1 | 6,25% | 3 | 5,77% |
| 3- partly agree | 2 | 11% | 3 | 18% | 2 | 12,50% | 7 | 13,46% |
| 4- fully agree | 17 | 89% | 12 | 71% | 13 | 81,25% | 42 | 80,77% |
| Total | 19 | 100% | 17 | 100% | 16 | 100% | 52 | 100,00% |
| I will recommend this case study to my colleagues to learn more about the second victim phenomenon. | N | % | N | % | N | % | N | % |
| 1- fully disagree | - | - | - | - | - | - |  |  |
| 2- partly disagree | - | - | 2 | 11,80 | 1 | 6,25% | 3 | 5,77% |
| 3- partly agree | 3 | 16% | 1 | 5,90 | 1 | 6,25% | 5 | 9,6% |
| 4- fully agree | 16 | 84% | 14 | 82,40 | 14 | 87,50% | 44 | 84,62% |
| Total | 19 | 100% | 17 |  | 16 | 100% | 52 | 100% |
| Do you consider that the time for working group discussion was adequate to achieve the learning goals? | N | % | N | % | N | % | N | % |
| The time was too short | - | - | - | - | 1 | 6,25% | 1 | 1,92% |
| The time was longer than necessary | 1 | 5,26% | 1 | 5,90 | 1 | 6,25% | 3 | 5,77% |
| The time was adequate | 5 | 26,32% | 16 | 94,10 | 14 | 87,50% | 35 | 67,31% |
| Prefer not to answer | 13 | 68,42% |  |  |  |  | 13 | 25% |
| Total | 19 | 100% | 17 | 100 | 16 | 100% | 52 | 100,00% |
| Do you consider that the time to prepare the presentation of the main conclusions of the working group discussion was adequate? | N | % | N | % | N | % | N | % |
| The time was to short | - | - | - | - | 5 | 31,25% | 5 | 9,62% |
| The time was longer than necessary | 1 | 5,26% | 1 | 5,90 | - | - | 2 | 3,85% |
| The time was adequate | 7 | 36,84% | 16 | 94,10 | - | - | 23 | *44,23%* |
| Prefer not to answer | *11* | 57,89% |  |  | 11 | 68,75% | 22 | 42,31% |
| Total | 19 | 100% | 17 | 100 | 16 | 100% | 52 | 100,00% |
| Do you consider that the time for presentation of the main conclusion of the working groups in the plenary session was adequate? | N | % | N | % | N | % | N | % |
| The time was to short | - | - | 2 | 11,80 | - | - | 2 | 3,85% |
| The time was longer than necessary | - | - | - | - | 1 | 6,25% | 1 | 1,92% |
| The time was adequate | 12 | 63,16% | 15 | 88,20 | 15 | 93,75% | 42 | 80,77% |
| Prefer not to answer | 7 | 36,84% | - | - | - |  | 7 | 13,46% |
| Total | 19 | 100% | 17 | 100 | 16 | 100% | 52 | 100,00% |
| Do you consider that the method used for discussion (roundtable in working groups) was adequate to achieve the learning goals? | N | % | N | % | N | % | N | % |
| The method was adequate | 17 | 89,47% | 13 | 76,50 | 14 | 87,50% | 44 | 84,62% |
| The method was satisfactory, however was not the most adequate to achieve the leaning goals | 2 | 10,53% | 4 | 23,50 | 1 | 6,25% | 7 | 13,46% |
| The method was not adequate at all | - | - | - | - | 1 | 6,25% | 1 | 1,92% |
| Prefer not to answer | - | - | - | - | - | - | 0 | 0 |
| Total | 19 | 100% | 17 | 100 | 16 | 100% | 52 | 100,00% |

Table S10- Evaluation of cases studies 2^nd^ edition

| Responses | Case study 1 | | Case study 2 | | Case study 3 | | Total | |
| --- | --- | --- | --- | --- | --- | --- | --- | --- |
| The leaning goals were clear | N | % | N | % | N | % | N | % |
| 1- fully disagree | - | - | - | - | - | - | - | - |
| 2- partly disagree | 1 | 6% | 1 | 5% | - | - | 2 | 3,57% |
| 3- partly agree | 4 | 22% | 4 | 21% | 4 | 31,6 | 12 | 21,43% |
| 4- fully agree | 13 | 72% | 14 | 74% | 15 | 68,4 | 42 | 75,00% |
|  | 18 | 100% | 19 | 100% | 19 | 100 | 56 | 100% |
| The scenario was realistic comparing to the current healthcare practice | N | % | N | % | N | % | N | % |
| 1- fully disagree | 1 | 5,56% | - | - | - | - |  |  |
| 2- partly disagree | 1 | 5,56% | - | - | - | - | 2 | 3,57% |
| 3- partly agree | 6 | 33,33% | 1 | 5,26% | 2 | 10,53% | 9 | 16,07% |
| 4- fully agree | 10 | 55,56% | 18 | 94,74% | 17 | 89,47% | 45 | 80,36% |
|  | 18 | 100,00% | 19 | 100,00% | 19 | 100,00% | 56 | 100,00% |
| The content of the case study was clear | N | % | N | % | N | % | N | % |
| 1- fully disagree | 0 | 5,56% | - | - |  |  | 0 | 0,00% |
| 2- partly disagree | 1 | 5,56% | - | - |  |  | 1 | 1,79% |
| 3- partly agree | 4 | 27,78% | 3 | 15,79% | 3 | 15,79% | 10 | 17,86% |
| 4- fully agree | 13 | 61,11% | 16 | 84,21% | 16 | 84,21% | 45 | 80,36% |
|  | 18 | 100% | 19 | 100,00% | 19 | 100,00% | 56 | 100,00% |
| The supporting information of the case studies (explanations/examples/other sources suggestions) was clear. | N | % | N | % | N | % | N | % |
| 1- fully disagree | 0 | 0,00% | - | - | - | - | 0 | 0,00% |
| 2- partly disagree | 1 | 5,56% | 1 | 5,26% | - | - | 2 | 5,41% |
| 3- partly agree | 4 | 22,22% | 1 | 5,26% | - | - | 5 | 13,51% |
| 4- fully agree | 13 | 72,22% | 17 | 89,47% | - | - | 30 | 81,08% |
|  | 18 | 100,00% | 19 | 100,00% |  |  | 37 | 100,00% |
| The knowledge obtained from the case study will (positively) affect my daily practice. | N | % | N | % | N | % | N | % |
| 1- fully disagree | - | - | - | - | - | - | - | - |
| 2- partly disagree | 1 | 5,56% | - | - | - | - | 1 | 1,79% |
| 3- partly agree | 5 | 27,78% | 5 | 26,32% | 6 | 31,58% | 16 | 28,57% |
| 4- fully agree | 12 | 66,67% | 14 | 73,68% | 13 | 68,42% | 39 | 69,64% |
|  | 18 | 100,00% | 19 |  | 19 |  | 56 | 100,00% |
| I will recommend this case study to my colleagues to learn more about the second victim phenomenon. | N | % | N | % | N | % | N | % |
| 1- fully disagree | 1 | 5,56% | - | - | - | - | 1 | 1,79% |
| 2- partly disagree | 1 | 5,56% | - | - | - | - | 1 | 1,79% |
| 3- partly agree | 7 | 38,89% | 3 | 15,79% | 3 | 15,79% | 13 | 23,21% |
| 4- fully agree | 9 | 50,00% | 16 | 84,21% | 16 | 84,21% | 41 | 73,21% |
|  | 18 |  | 19 |  | 19 |  | 56 | 100,00% |
| Do you consider that the time for working group discussion was adequate to achieve the learning goals? | N | % | N | % | N | % | N | % |
| The time was too short | 3 | 16,67% | 2 | 10,53% | 2 | 10,53% | 7 | 12,50% |
| The time was longer than necessary | 1 | 5,56% | 1 | 5,26% | - | - | 2 | 3,57% |
| The time was adequate | 14 | 77,78% | 16 | 84,21% | 17 | 89,47% | 47 | 83,93% |
| Prefer not to answer | - | - | - | - | - | - | 0 | 0,00% |
|  | 18 | 1 | 19 |  | 19 |  | 56 | 100,00% |
| Do you consider that the time to prepare the presentation of the main conclusions of the working group discussion was adequate? | N | % | N | % | N | % | N | % |
| The time was to short | 2 | 11,11% | 2 | 10,53% | 3 | 15,79% | 7 | 12,50% |
| The time was longer than necessary | - | - | 1 | 5,26% | - | - | 1 | 1,79% |
| The time was adequate | 16 | 88,89% | 16 | 84,21% | 16 | 84,21% | 48 | 85,71% |
| Prefer not to answer | - | - | 0 |  | - | - | 0 | 0 |
|  | 18 | 1 | 19 |  | 19 |  | 56 | 100,00% |
| Do you consider that the time for presentation of the main conclusion of the working groups in the plenary session was adequate? | N | % | N | % | N | % | N | % |
| The time was to short | 2 | 11,11% | 2 | 10,53% | 1 | 5,26% | 5 | 8,93% |
| The time was longer than necessary | 2 | 11,11% | 1 | 5,26% | 2 | 10,53% | 5 | 8,93% |
| The time was adequate | 14 | 77,78% | 16 | 84,21% | 16 | 84,21% | 46 | 82,14% |
| Prefer not to answer | - | - | - | - | - | - | 0 | 0,00% |
|  | 18 | 1 | 19 |  | 19 |  | 56 | 100,00% |
|  | Case study 1 | | Case study 2 | | Case study 3 | |  |  |
| Do you consider that the method used for discussion (roundtable in working groups) was adequate to achieve the learning goals? | N | % | N | % | N | % | N | % |
| The method was adequate | 15 | 83,33% | 15 | 78,95% | 15 | 78,94% | 45 | 80,36% |
| The method was satisfactory, however was not the most adequate to achieve the leaning goals | 2 | 11,11% | 2 | 10,53% | 3 | 15,79% | 7 | 12,50% |
| The method was not adequate at all | 1 | 5,56% | 1 | 5,26% | 1 | 5,26% | 3 | 5,36% |
| Prefer not to answer | - | - | 1 | 5,26% | - | - | 1 | 1,79% |
|  | 18 |  | 19 |  | 19 |  | 56 | 100,00% |

Table S11– Comparison of evaluation of cases studies of the 1^st^ and 2^nd^ edition

|  | 1st edition Total | |  | 2nd Edition Total | | P value |
| --- | --- | --- | --- | --- | --- | --- |
| The leaning goals were clear | N | % | N |  | % |  |
| 1- fully disagree |  |  | - |  | - |  |
| 2- partly disagree | 2 | 3,85% | 2 |  | 3,57% |  |
| 3- partly agree | 6 | 11,54% | 12 |  | 21,43% |  |
| 4- fully agree | 44 | 84,62% | 42 |  | 75,00% |  |
|  | 52 | 100,00% | 56 |  | 100% | p=0,349 |
| The scenario was realistic comparing to the current healthcare practice | N | % | N |  | % |  |
| 1- fully disagree | 0 | 0% | 0 |  | 0,00% |  |
| 2- partly disagree | 2 | 3,85% | 2 |  | 3,57% |  |
| 3- partly agree | 4 | 7,69% | 9 |  | 16,07% |  |
| 4- fully agree | 46 | 88,46% | 45 |  | 80,36% |  |
|  | 52 | 100,00% | 56 |  | 100,00% | p= 0.364 |
| The content of the case study was clear | N | % | N |  | % |  |
| 1- fully disagree |  |  | 0 |  |  |  |
| 2- partly disagree |  |  | 1 |  | 1,79% |  |
| 3- partly agree | 6 | 11,54% | 10 |  | 17,86% |  |
| 4- fully agree | 46 | 88,46% | 45 |  | 80,36% |  |
|  | 52 | 100,00% | 56 |  | 100,00% | p=0.418 |
| The supporting information of the case studies (explanations/examples/other sources suggestions) was clear. | N | % | N |  | % |  |
| 1- fully disagree | 0 | 0,00% | 0 |  | 0,00% |  |
| 2- partly disagree | 1 | 2,78% | 2 |  | 5,41% |  |
| 3- partly agree | 7 | 19,44% | 5 |  | 13,51% |  |
| 4- fully agree | 28 | 77,78% | 30 |  | 81,08% |  |
|  | 36 | 100,00% | 37 |  | 100,00% | p=0.729 |
| The knowledge obtained from the case study will (positively) affect my daily practice. | N | % | N |  | % |  |
| 1- fully disagree | 0 | 0,00% | c |  | 0,00% |  |
| 2- partly disagree | 3 | 5,77% | 1 |  | 1,79% |  |
| 3- partly agree | 7 | 13,46% | 16 |  | 28,57% |  |
| 4- fully agree | 42 | 80,77% | 39 |  | 69,64% |  |
|  | 52 | 100,00% | 56 |  | 100,00% | p=0.107 |
| I will recommend this case study to my colleagues to learn more about the second victim phenomenon. | N | % | N |  | % |  |
| 1- fully disagree |  |  | 1 |  | 1,79% |  |
| 2- partly disagree | 3 | 5,77% | 1 |  | 1,79% |  |
| 3- partly agree | 5 | 9,6% | 13 |  | 23,21% |  |
| 4- fully agree | 44 | 84,62% | 41 |  | 73,21% |  |
|  | 52 | 100% | 56 |  | 100,00% | p=0.113 |
| Do you consider that the time for working group discussion was adequate to achieve the learning goals? | N | % | N |  | % |  |
| The time was too short | 1 | 1,92% | 7 |  | 12,50% |  |
| The time was longer than necessary | 3 | 5,77% | 2 |  | 3,57% |  |
| The time was adequate | 35 | 67,31% | 47 |  | 83,93% |  |
| Prefer not to answer | 13 | 25% | 0 |  | 0,00% |  |
|  | 52 | 100,00% | 56 |  | 100,00% | p<.001 |
| Do you consider that the time to prepare the presentation of the main conclusions of the working group discussion was adequate? | N | % | N |  | % |  |
| The time was to short | 5 | 9,62% | 7 |  | 12,50% |  |
| The time was longer than necessary | 2 | 3,85% | 1 |  | 1,79% |  |
| The time was adequate | 23 | 44,23% | 48 |  | 85,71% |  |
| Prefer not to answer | 22 | 42,31% | 0 |  | 0 |  |
|  | 52 | 100,00% | 56 |  | 100,00% | p<.001 |
| Do you consider that the time for presentation of the main conclusion of the working groups in the plenary session was adequate? | N | % | N |  | % |  |
| The time was to short | 2 | 3,85% | 5 |  | 8,93% |  |
| The time was longer than necessary | 1 | 1,92% | 5 |  | 8,93% |  |
| The time was adequate | 42 | 80,77% | 46 |  | 82,14% |  |
| Prefer not to answer | 7 | 13,46% | 0 |  | 0,00% |  |
|  | 52 | 100,00% | 56 |  | 100,00% | P=0.006 |
| Do you consider that the method used for discussion (roundtable in working groups) was adequate to achieve the learning goals? | N | % | N |  | % |  |
| The method was adequate | 44 | 84,62% | 45 |  | 80,36% |  |
| The method was satisfactory, however was not the most adequate to achieve the leaning goals | 7 | 13,46% | 7 |  | 12,50% |  |
| The method was not adequate at all | 1 | 1,92% | 3 |  | 5,36% |  |
| Prefer not to answer | 0 | 0 |  |  | 0 |  |
|  | 52 | 100,00% | 1 |  | 100,00% | p=0.772 |

Table S12- evaluation of the Second Victim Podcast

| 1st edition |  |  | 2nd edition | | P value |
| --- | --- | --- | --- | --- | --- |
| Podcast was useful for case study discussion | N | % | N | % | p=0.265 |
| Partially disagree | 1 | 8,33% | 0 | 0% |  |
| Partially agree | 4 | 33,33% | 3 | 17,65% |  |
| Fully agree | 7 | 58,33% | 14 | 82,35% |  |
| Total | 12 | 100,00% | 17 | 100,00% |  |

Table S13- evaluation of the preconference included in the 2^nd^ edition of the Training School

| I found the Pre Conference useful | N | % |
| --- | --- | --- |
| 1- fully disagree | 1 | 5,26% |
| 2- partly disagree |  |  |
| 3- partly agree |  |  |
| 4- fully agree | 18 | 94,74% |
| Total | 19 | 100,00% |
| The period of time of the online event was adequated | N | % |
| 1- fully disagree | 1 | 5,26% |
| 2- partly disagree |  | 0,00% |
| 3- partly agree | 6 | 31,58% |
| 4- fully agree | 12 | 63,16% |
| Total | 19 | 100,00% |
| I found the content of the sessions important | N | % |
| 1- fully disagree | 1 | 5,26% |
| 2- partly disagree |  |  |
| 3- partly agree | 4 | 21,05% |
| 4- fully agree | 14 | 73,68% |
| Total | 19 | 100,00% |
| After the Pre conference… | N/Total | % |
| The sessions helped to understand the overall goals of the Training School | 13/19 | 68% |
| The pre conference was important to get more involved in the Training School | 12/19 | 63% |
| I felt it was important to meet the trainers and other trainees | 15/19 | 79% |
| I feel more confident to follow the steps for e-cost reimbursement | 9/19 | 47% |
| I feel more motivated to attend the Training School after participating in the pre conference | 15/19 | 79% |
| I feel more confident after knowing other participants’ profiles | 2/19 | 11% |
