# Supplementary material for: Application and Evaluation of a Multimodal Training on the Second Victim Phenomenon at the European Researchers’ Network Working on Second Victims Training School: Mixed Methods Study
Source: JMIR Form Res. 2024 Aug 30;8:e58727. doi: 10.2196/58727 (PMC11418314; doi:10.2196/58727)
Supplement: Multimedia Appendix 7 [file formative_v8i1e58727_app7.docx]

**Multimedia Appendix 7**

**SPSS software (version 19.0.1.1; IBM Corp) output.**

1. **Responses to the online questionnaire**

**1.1. Question - The leaning goals were clear**

| **Chi-Square Tests** | | | | | | | | | |
| --- | --- | --- | --- | --- | --- | --- | --- | --- | --- |
|  | Value | df | Asymptotic Significance (2-sided) | Monte Carlo Sig. (2-sided) | | | Monte Carlo Sig. (1-sided) | | |
|  |  |  |  | Significance | 99% Confidence Interval | | Significance | 99% Confidence Interval | |
|  |  |  |  |  | Lower Bound | Upper Bound |  | Lower Bound | Upper Bound |
| Pearson Chi-Square | 1.901^a^ | 2 | .387 | .344^b^ | .332 | .357 |  |  |  |
| Likelihood Ratio | 1.937 | 2 | .380 | .344^b^ | .332 | .357 |  |  |  |
| Fisher-Freeman-Halton Exact Test | 1.980 |  |  | .344^b^ | .332 | .357 |  |  |  |
| Linear-by-Linear Association | .907^c^ | 1 | .341 | .356^b^ | .343 | .368 | .221^b^ | .211 | .232 |
| N of Valid Cases | 108 |  |  |  |  |  |  |  |  |
| a. 2 cells (33.3%) have expected count less than 5. The minimum expected count is 1.93. | | | | | | | | | |
| b. Based on 10000 sampled tables with starting seed 2000000. | | | | | | | | | |
| c. The standardised statistic is -.953. | | | | | | | | | |

**1.2 Question –** The scenario was realistic comparing to the current healthcare practice

| **Chi-Square Tests** | | | | | | | | | |
| --- | --- | --- | --- | --- | --- | --- | --- | --- | --- |
|  | Value | df | Asymptotic Significance (2-sided) | Monte Carlo Sig. (2-sided) | | | Monte Carlo Sig. (1-sided) | | |
|  |  |  |  | Significance | 99% Confidence Interval | | Significance | 99% Confidence Interval | |
|  |  |  |  |  | Lower Bound | Upper Bound |  | Lower Bound | Upper Bound |
| Pearson Chi-Square | 1.788^a^ | 2 | .409 | .405^b^ | .392 | .417 |  |  |  |
| Likelihood Ratio | 1.836 | 2 | .399 | .405^b^ | .392 | .417 |  |  |  |
| Fisher-Freeman-Halton Exact Test | 1.852 |  |  | .364^b^ | .351 | .376 |  |  |  |
| Linear-by-Linear Association | .710^c^ | 1 | .400 | .434^b^ | .422 | .447 | .263^b^ | .252 | .274 |
| N of Valid Cases | 108 |  |  |  |  |  |  |  |  |
| a. 2 cells (33.3%) have expected count less than 5. The minimum expected count is 1.93. | | | | | | | | | |
| b. Based on 10000 sampled tables with starting seed 957002199. | | | | | | | | | |
| c. The standardised statistic is -.843. | | | | | | | | | |

**1.3 Question -** The content of the case study was clear

| **Chi-Square Tests** | | | | | | | | | | |
| --- | --- | --- | --- | --- | --- | --- | --- | --- | --- | --- |
|  | Value | df | Asymptotic Significance (2-sided) | Monte Carlo Sig. (2-sided) | | | Monte Carlo Sig. (1-sided) | | | |
|  |  |  |  | Significance | 99% Confidence Interval | | Significance | 99% Confidence Interval | |  |
|  |  |  |  |  | Lower Bound | Upper Bound |  | Lower Bound | Upper Bound |  |
| Pearson Chi-Square | 1.865^a^ | 2 | .393 | .418^b^ | .405 | .430 |  |  |  |  |
| Likelihood Ratio | 2.260 | 2 | .323 | .418^b^ | .405 | .430 |  |  |  |  |
| Fisher-Freeman-Halton Exact Test | 1.783 |  |  | .418^b^ | .405 | .430 |  |  |  |  |
| Linear-by-Linear Association | 1.660^c^ | 1 | .198 | .228^b^ | .217 | .239 | .145^b^ | .136 | .154 |  |
| N of Valid Cases | 108 |  |  |  |  |  |  |  |  |  |
| a. 2 cells (33.3%) have expected count less than 5. The minimum expected count is .48. | | | | | | | | | | |
| b. Based on 10000 sampled tables with starting seed 79654295. | | | | | | | | | | |
| c. The standardised statistic is -1.288. | | | | | | | | | | |

1.4 **Question -**The supporting information of the case studies (explanations/examples/other sources suggestions) was clear.

| **SUPPORTINF * EDITION Crosstabulation** | | | | |
| --- | --- | --- | --- | --- |
| Count | | | | |
|  | | EDITION | | Total |
|  |  | 1st edition | 2nd edition |  |
| SUPPORTINF | partially disagree | 1 | 2 | 3 |
|  | partially agree | 7 | 5 | 12 |
|  | fully agree | 28 | 30 | 58 |
| Total | | 36 | 37 | 73 |

| **Chi-Square Tests** | | | | | | | | | | |
| --- | --- | --- | --- | --- | --- | --- | --- | --- | --- | --- |
|  | Value | df | Asymptotic Significance (2-sided) | Monte Carlo Sig. (2-sided) | | | Monte Carlo Sig. (1-sided) | | |  |
|  |  |  |  | Significance | 99% Confidence Interval | | Significance | 99% Confidence Interval | |  |
|  |  |  |  |  | Lower Bound | Upper Bound |  | Lower Bound | Upper Bound |  |
| Pearson Chi-Square | .722^a^ | 2 | .697 | .729^b^ | .718 | .741 |  |  |  |  |
| Likelihood Ratio | .730 | 2 | .694 | .729^b^ | .718 | .741 |  |  |  |  |
| Fisher-Freeman-Halton Exact Test | .818 |  |  | .729^b^ | .718 | .741 |  |  |  |  |
| Linear-by-Linear Association | .003^c^ | 1 | .956 | 1.000^b^ | 1.000 | 1.000 | .557^b^ | .544 | .570 |  |
| N of Valid Cases | 73 |  |  |  |  |  |  |  |  |  |
| a. 2 cells (33.3%) have expected count less than 5. The minimum expected count is 1.48. | | | | | | | | | | |
| b. Based on 10000 sampled tables with starting seed 1993510611. | | | | | | | | | | |
| c. The standardised statistic is .055. | | | | | | | | | | |

1.5 Question - The knowledge obtained from the case study will (positively) affect my daily practice.

| **IMPACT PRACTICE * EDITION Crosstabulation** | | | | |
| --- | --- | --- | --- | --- |
| Count | | | | |
|  | | EDITION | | Total |
|  |  | 1st edition | 2nd edition |  |
| PRACTICE | Partially disagree | 3 | 1 | 4 |
|  | Partially agree | 7 | 16 | 23 |
|  | Fully agree | 42 | 39 | 81 |
| Total | | 52 | 56 | 108 |

| **Chi-Square Tests** | | | | | | | | | |
| --- | --- | --- | --- | --- | --- | --- | --- | --- | --- |
|  | Value | df | Asymptotic Significance (2-sided) | Monte Carlo Sig. (2-sided) | | | Monte Carlo Sig. (1-sided) | | |
|  |  |  |  | Significance | 99% Confidence Interval | | Significance | 99% Confidence Interval | |
|  |  |  |  |  | Lower Bound | Upper Bound |  | Lower Bound | Upper Bound |
| Pearson Chi-Square | 4.491^a^ | 2 | .106 | .127^b^ | .118 | .135 |  |  |  |
| Likelihood Ratio | 4.627 | 2 | .099 | .172^b^ | .162 | .181 |  |  |  |
| Fisher-Freeman-Halton Exact Test | 4.380 |  |  | .107^b^ | .099 | .115 |  |  |  |
| Linear-by-Linear Association | .489^c^ | 1 | .484 | .584^b^ | .572 | .597 | .304^b^ | .292 | .315 |
| N of Valid Cases | 108 |  |  |  |  |  |  |  |  |
| a. 2 cells (33.3%) have expected count less than 5. The minimum expected count is 1.93. | | | | | | | | | |
| b. Based on 10000 sampled tables with starting seed 2000000. | | | | | | | | | |
| c. The standardised statistic is -.699. | | | | | | | | | |

1.6- Question 6- I will recommend this case study to my colleagues to learn more about the second victim phenomenon.

| **Recommend * EDITION Crosstabulation** | | | | |
| --- | --- | --- | --- | --- |
| Count | | | | |
|  | | EDITION | | Total |
|  |  | 1 | 2 |  |
| Recommend | fully disagree | 0 | 1 | 1 |
|  | partially disagree | 3 | 1 | 4 |
|  | partially agree | 5 | 13 | 18 |
|  | fully agree | 44 | 41 | 85 |
| Total | | 52 | 56 | 108 |

| **Chi-Square Tests** | | | | | | | | | | |
| --- | --- | --- | --- | --- | --- | --- | --- | --- | --- | --- |
|  | Value | df | Asymptotic Significance (2-sided) | Monte Carlo Sig. (2-sided) | | | Monte Carlo Sig. (1-sided) | | | |
|  |  |  |  | Significance | 99% Confidence Interval | | Significance | 99% Confidence Interval | |  |
|  |  |  |  |  | Lower Bound | Upper Bound |  | Lower Bound | Upper Bound |  |
| Pearson Chi-Square | 5.521^a^ | 3 | .137 | .117^b^ | .109 | .125 |  |  |  |  |
| Likelihood Ratio | 6.073 | 3 | .108 | .169^b^ | .159 | .178 |  |  |  |  |
| Fisher-Freeman-Halton Exact Test | 5.332 |  |  | .113^b^ | .104 | .121 |  |  |  |  |
| Linear-by-Linear Association | .989^c^ | 1 | .320 | .409^b^ | .396 | .422 | .210^b^ | .200 | .220 |  |
| N of Valid Cases | 108 |  |  |  |  |  |  |  |  |  |
| a. 4 cells (50.0%) have expected count less than 5. The minimum expected count is .48. | | | | | | | | | | |
| b. Based on 10000 sampled tables with starting seed 624387341. | | | | | | | | | | |
| c. The standardised statistic is -.995. | | | | | | | | | | |

- 1. Question 7- Do you consider that the time for working group discussion was adequate to achieve the learning goals?

| **TIME for WG Discussion * EDITION Crosstabulation** | | | | |
| --- | --- | --- | --- | --- |
| Count | | | | |
|  | | EDITION | | Total |
|  |  | 1.00 | 2.00 |  |
| TIMEDISC | The time was too short | 1 | 7 | 8 |
|  | Time was longer than necessary | 3 | 2 | 5 |
|  | Time was adequate | 35 | 47 | 82 |
|  | Prefer not to answer | 13 | 0 | 13 |
| Total | | 52 | 56 | 108 |

| **Chi-Square Tests** | | | | | | | | | | |
| --- | --- | --- | --- | --- | --- | --- | --- | --- | --- | --- |
|  | Value | df | Asymptotic Significance (2-sided) | Monte Carlo Sig. (2-sided) | | | Monte Carlo Sig. (1-sided) | | |  |
|  |  |  |  | Significance | 99% Confidence Interval | | Significance | 99% Confidence Interval | |  |
|  |  |  |  |  | Lower Bound | Upper Bound |  | Lower Bound | Upper Bound |  |
| Pearson Chi-Square | 19.334^a^ | 3 | <.001 | <.001^b^ | <.001 | <.001 |  |  |  |  |
| Likelihood Ratio | 24.899 | 3 | <.001 | <.001^b^ | <.001 | <.001 |  |  |  |  |
| Fisher-Freeman-Halton Exact Test | 21.271 |  |  | <.001^b^ | <.001 | <.001 |  |  |  |  |
| Linear-by-Linear Association | 11.282^c^ | 1 | <.001 | <.001^b^ | <.001 | .002 | <.001^b^ | <.001 | <.001 |  |
| N of Valid Cases | 108 |  |  |  |  |  |  |  |  |  |
| a. 4 cells (50.0%) have expected count less than 5. The minimum expected count is 2.41. | | | | | | | | | | |
| b. Based on 10000 sampled tables with starting seed 957002199. | | | | | | | | | | |
| c. The standardised statistic is -3.359. | | | | | | | | | | |

- 1. Question 8- Do you consider that the time to prepare the presentation of the main conclusions of the working group discussion was adequate?

| **TIME Presentation Preparation * EDITION Crosstabulation** | | | | |
| --- | --- | --- | --- | --- |
| Count | | | | |
|  | | EDITION | | Total |
|  |  | 1.00 | 2.00 |  |
| TIMEPREP | The time was to short | 5 | 7 | 12 |
|  | The time was longer than necessary | 2 | 1 | 3 |
|  | The time was adequate | 23 | 48 | 71 |
|  | Prefer not to answer | 22 | 0 | 22 |
| Total | | 52 | 56 | 108 |

| **Chi-Square Tests** | | | | | | | | | |
| --- | --- | --- | --- | --- | --- | --- | --- | --- | --- |
|  | Value | df | Asymptotic Significance (2-sided) | Monte Carlo Sig. (2-sided) | | | Monte Carlo Sig. (1-sided) | | |
|  |  |  |  | Significance | 99% Confidence Interval | | Significance | 99% Confidence Interval | |
|  |  |  |  |  | Lower Bound | Upper Bound |  | Lower Bound | Upper Bound |
| Pearson Chi-Square | 31.364^a^ | 3 | <.001 | <.001^b^ | <.001 | <.001 |  |  |  |
| Likelihood Ratio | 40.019 | 3 | <.001 | <.001^b^ | <.001 | <.001 |  |  |  |
| Fisher-Freeman-Halton Exact Test | 36.440 |  |  | <.001^b^ | <.001 | <.001 |  |  |  |
| Linear-by-Linear Association | 8.395^c^ | 1 | .004 | .004^b^ | .003 | .006 | .002^b^ | <.001 | .003 |
| N of Valid Cases | 108 |  |  |  |  |  |  |  |  |
| a. 2 cells (25.0%) have expected count less than 5. The minimum expected count is 1.44. | | | | | | | | | |
| b. Based on 10000 sampled tables with starting seed 92208573. | | | | | | | | | |
| c. The standardised statistic is -2.897. | | | | | | | | | |

- 1. Question 9- Do you consider that the time for presentation of the main conclusion of the working groups in the plenary session was adequate?

| **TimePresent * Edition Crosstabulation** | | | | |
| --- | --- | --- | --- | --- |
| Count | | | | |
|  | | Edition | | Total |
|  |  | 1.00 | 2.00 |  |
| TimePresent | The time was to short | 2 | 5 | 7 |
|  | The time was longer than necessary | 1 | 5 | 6 |
|  | The time was adequate | 42 | 46 | 88 |
|  | Prefer not to answer | 7 | 0 | 7 |
| Total | | 52 | 56 | 108 |

| **Chi-Square Tests** | | | | | | | | | |
| --- | --- | --- | --- | --- | --- | --- | --- | --- | --- |
|  | Value | df | Asymptotic Significance (2-sided) | Monte Carlo Sig. (2-sided) | | | Monte Carlo Sig. (1-sided) | | |
|  |  |  |  | Significance | 99% Confidence Interval | | Significance | 99% Confidence Interval | |
|  |  |  |  |  | Lower Bound | Upper Bound |  | Lower Bound | Upper Bound |
| Pearson Chi-Square | 11.001^a^ | 3 | .012 | .007^b^ | .005 | .009 |  |  |  |
| Likelihood Ratio | 13.977 | 3 | .003 | .007^b^ | .005 | .009 |  |  |  |
| Fisher-Freeman-Halton Exact Test | 11.053 |  |  | .007^b^ | .005 | .009 |  |  |  |
| Linear-by-Linear Association | 6.865^c^ | 1 | .009 | .010^b^ | .007 | .012 | .005^b^ | .004 | .007 |
| N of Valid Cases | 108 |  |  |  |  |  |  |  |  |
| a. 6 cells (75.0%) have expected count less than 5. The minimum expected count is 2.89. | | | | | | | | | |
| b. Based on 10000 sampled tables with starting seed 475497203. | | | | | | | | | |
| c. The standardised statistic is -2.620. | | | | | | | | | |

- 1. Question 10- Do you consider that the method used for discussion (roundtable in working groups) was adequate to achieve the learning goals?

| **Method * Edition Crosstabulation** | | | | |
| --- | --- | --- | --- | --- |
| Count | | | | |
|  | | Edition | | Total |
|  |  | 1.00 | 2.00 |  |
| Method | The method was adequate | 44 | 45 | 89 |
|  | The method was satisfactory, however was not the most adequate to achieve the leaning goals | 7 | 7 | 14 |
|  | The method was not adequate at all | 1 | 3 | 4 |
| Total | | 52 | 55 | 107 |

| **Chi-Square Tests** | | | | | | | | | |
| --- | --- | --- | --- | --- | --- | --- | --- | --- | --- |
|  | Value | df | Asymptotic Significance (2-sided) | Monte Carlo Sig. (2-sided) | | | Monte Carlo Sig. (1-sided) | | |
|  |  |  |  | Significance | 99% Confidence Interval | | Significance | 99% Confidence Interval | |
|  |  |  |  |  | Lower Bound | Upper Bound |  | Lower Bound | Upper Bound |
| Pearson Chi-Square | .928^a^ | 2 | .629 | .772^b^ | .762 | .783 |  |  |  |
| Likelihood Ratio | .974 | 2 | .615 | .772^b^ | .762 | .783 |  |  |  |
| Fisher-Freeman-Halton Exact Test | .901 |  |  | .772^b^ | .762 | .783 |  |  |  |
| Linear-by-Linear Association | .445^c^ | 1 | .505 | .569^b^ | .556 | .582 | .322^b^ | .310 | .334 |
| N of Valid Cases | 107 |  |  |  |  |  |  |  |  |
| a. 2 cells (33.3%) have expected count less than 5. The minimum expected count is 1.94. | | | | | | | | | |
| b. Based on 10000 sampled tables with starting seed 1122541128. | | | | | | | | | |
| c. The standardised statistic is .667. | | | | | | | | | |

1. Podcast evaluation

| **podcast * Edition Crosstabulation** | | | | |
| --- | --- | --- | --- | --- |
| Count | | | | |
|  | | Edition | | Total |
|  |  | 1.00 | 2.00 |  |
| podcast | partially disagree | 1 | 0 | 1 |
|  | partially agree | 4 | 3 | 7 |
|  | fully agree | 7 | 14 | 21 |
| Total | | 12 | 17 | 29 |
|  | |  |  |  |

| **Chi-Square Tests** | | | | | | | | | |
| --- | --- | --- | --- | --- | --- | --- | --- | --- | --- |
|  | Value | df | Asymptotic Significance (2-sided) | Monte Carlo Sig. (2-sided) | | | Monte Carlo Sig. (1-sided) | | |
|  |  |  |  | Significance | 99% Confidence Interval | | Significance | 99% Confidence Interval | |
|  |  |  |  |  | Lower Bound | Upper Bound |  | Lower Bound | Upper Bound |
| Pearson Chi-Square | 2.694^a^ | 2 | .260 | .265^b^ | .254 | .277 |  |  |  |
| Likelihood Ratio | 3.042 | 2 | .219 | .265^b^ | .254 | .277 |  |  |  |
| Fisher-Freeman-Halton Exact Test | 2.625 |  |  | .265^b^ | .254 | .277 |  |  |  |
| Linear-by-Linear Association | 2.512^c^ | 1 | .113 | .163^b^ | .154 | .173 | .107^b^ | .099 | .115 |
| N of Valid Cases | 29 |  |  |  |  |  |  |  |  |
| a. 4 cells (66.7%) have expected count less than 5. The minimum expected count is .41. | | | | | | | | | |
| b. Based on 10000 sampled tables with starting seed 957002199. | | | | | | | | | |
| c. The standardised statistic is 1.585. | | | | | | | | | |

1. Supplementary information - case study 1 evaluation between 1^st^ and 2^nd^ edition

Question - The supporting information of the case studies (explanations/examples/other sources suggestions) was clear.

| **CASESTUDY1 * EDITION Crosstabulation** | | | | |
| --- | --- | --- | --- | --- |
| Count | | | | |
|  | | EDITION | | Total |
|  |  | 1st edition | 2nd edition |  |
| CASESTUDY1 | partially disagree | 0 | 1 | 1 |
|  | partially agree | 7 | 4 | 11 |
|  | fully agree | 12 | 13 | 25 |
| Total | | 19 | 18 | 37 |

| **Chi-Square Tests** | | | | | | | | | |
| --- | --- | --- | --- | --- | --- | --- | --- | --- | --- |
|  | Value | df | Asymptotic Significance (2-sided) | Monte Carlo Sig. (2-sided) | | | Monte Carlo Sig. (1-sided) | | |
|  |  |  |  | Significance | 99% Confidence Interval | | Significance | 99% Confidence Interval | |
|  |  |  |  |  | Lower Bound | Upper Bound |  | Lower Bound | Upper Bound |
| Pearson Chi-Square | 1.832^a^ | 2 | .400 | .475^b^ | .462 | .487 |  |  |  |
| Likelihood Ratio | 2.228 | 2 | .328 | .475^b^ | .462 | .487 |  |  |  |
| Fisher-Freeman-Halton Exact Test | 1.756 |  |  | .475^b^ | .462 | .487 |  |  |  |
| Linear-by-Linear Association | .039^c^ | 1 | .843 | 1.000^b^ | 1.000 | 1.000 | .542^b^ | .529 | .555 |
| N of Valid Cases | 37 |  |  |  |  |  |  |  |  |
| a. 2 cells (33.3%) have expected count less than 5. The minimum expected count is .49. | | | | | | | | | |
| b. Based on 10000 sampled tables with starting seed 475497203. | | | | | | | | | |
| c. The standardised statistic is .198. | | | | | | | | | |
